# Autophagic Schwann cells promote perineural invasion mediated by the NGF/ATG7 paracrine pathway in pancreatic cancer

**DOI:** 10.1186/s13046-021-02198-w

**Published:** 2022-02-02

**Authors:** Wunai Zhang, Rui He, Wenbin Yang, Yan Zhang, Qinggong Yuan, Jixin Wang, Yang Liu, Shuo Chen, Simei Zhang, Weifan Zhang, Zeen Zhu, Jing Zhang, Zheng Wang, Junhui Li

**Affiliations:** 1 Department of General Surgery, Second Affiliated Hospital, Xi’an Jiaotong University, 157 West 5th Road, Xi’an, 710004 China; 2 Department of Hepatobiliary Surgery, The First Affiliated Hospital of Xi’an Jiaotong University, 710061, Xi’an, Shaanxi Province China; 3 Department of Oncology, The Second Affiliated Hospital of Xi’an Jiaotong University, 157 West 5th Road, Xi’an, 710004 China; 4 Department of Anesthesiology, The Second Affiliated Hospital of Xi’an Jiaotong University, 157 West 5th Road, Xi’an, 710004 China

**Keywords:** Pancreatic cancer, Schwann cells, Autophagy, NGF, Perineural invasion

## Abstract

**Background:**

Perineural invasion (PNI) and autophagy are two common features in the tumor microenvironment of pancreatic cancer (PanCa) and have a negative effect on prognosis. Potential mediator cells and the molecular mechanism underlying their relationships need to be fully elucidated.

**Methods:**

To investigate the autophagy of Schwann cells (SCs) in PNI, we reproduced the microenvironment of PNI by collecting clinical PNI tissue, performing sciatic nerve injection of nude mice with cancer cells and establishing a Dorsal root ganglion (DRG) coculture system with cancer cell lines. Autophagy was detected by IHC, IF, transmission electron microscopy (TEM) and western blotting assays. Apoptosis was detected by IF, TEM and western blotting. NGF targeting molecular RO 08–2750(RO) and the autophagy inhibitor Chloroquine (CQ) were utilized to evaluate the effect on autophagy and apoptosis in SCs and PanCa cells in PNI samples.

**Results:**

SC autophagy is activated in PNI by paracrine NGF from PanCa cells. Autophagy-activated Schwann cells promote PNI through a) enhanced migration and axon guidance toward PanCa cells and b) increased chemoattraction to PanCa cells. The NGF-targeting reagent RO and autophagy inhibitor CQ inhibited Schwann cell autophagic flux and induced Schwann cell apoptosis. Moreover, RO and CQ could induce PanCa cell apoptosis and showed good therapeutic effects in the PNI model.

**Conclusions:**

PanCa cells can induce autophagy in SCs through paracrine pathways such as the NGF/ATG7 pathway. Autophagic SCs exert a “nerve-repair like effect”, induce a high level of autophagy of cancer cells, provide a “beacon” for the invasion of cancer cells to nerve fibers, and induce directional growth of cancer cells. Targeting NGF and autophagy for PNI treatment can block nerve infiltration and is expected to provide new directions and an experimental basis for the research and treatment of nerve infiltration in pancreatic cancer.

**Supplementary Information:**

The online version contains supplementary material available at 10.1186/s13046-021-02198-w.

## Background

Pancreatic ductal adenocarcinoma (PanCa) is a lethal disease with high morbidity and mortality worldwide. It was estimated that, by 2040, the total number of cases in the European Union (EU) will increase by more than 30% [1]. PanCa is the seventh and fourth leading cause of cancer-related deaths worldwide and in the United States, respectively [2, 3]. PanCa is projected to surpass breast, prostate, and colorectal cancers to become the second leading cause of cancer-related death by 2030 [4]. Perineural invasion (PNI), with a prevalence between 70 and 100% in PanCa, is associated with poor prognosis, tumor recurrence, and generation of pain; this condition can be detected in the early stages and is an independent prognostic factor of PanCa [5, 6].

Peripheral nerves form complex tumor microenvironments, which are comprised of several cell types and factors, including Schwann cells [7, 8]. Recent studies have revealed that Schwann cells (SCs) can enable cancer progression by adopting a dedifferentiated phenotype, which is similar to the SC response to nerve trauma [9]. A detailed understanding of the molecular and cellular mechanisms involved in the regulation of cancer progression by nerves is essential to design strategies to inhibit tumor progression [10]. Direct contact between cancer cells and Schwann cells can promote cancer cell migration, dissociation, and invasion. The Schwann cell-directed regulation of cancer cells may be mediated by neural cell adhesion molecule 1 (NCAM1) in perineural invasion [9]. Schwann cells also contribute to PanCa epithelial-mesenchymal transition (EMT) in pancreatic nerves by activating the MET pathway in cancer cells in the PanCa microenvironment [11]. SC-conditioned media can promote the proliferation and invasion of PanCa through matrix metalloproteinase-2, cathepsin D, plasminogen activator inhibitor-1, and galectin-1, as shown by proteomic analysis [12]. SCs are dedifferentiated and participated in axonal regeneration after peripheral nerve injury, which is critical to achieving efficient axonal regeneration at the early stages [13].

Pancreatic cancer primary tumors and cell lines show elevated autophagy under basal conditions. Autophagy was significantly induced in pancreatic ductal adenocarcinoma tissue compared to healthy pancreatic parenchyma of patients. Autophagy has a cytoprotective effect against 5-fluorouracil and gemcitabine in pancreatic cancer cells [14]. In SCs, autophagy is activated and exerts a ubiquitous cytoprotective effect, which is essential for degrading and recycling cellular constituents after nerve damage [15]. Nerve growth factor (NGF) may activate the autophagy of SCs, which is crucial for degradation and clearance of myelin debris following peripheral nerve injury via the p75NTR/AMPK/mTOR axis [16].

However, little is known about the effect and mechanism of cancer cells on SCs in the PNI of PanCa. In the present study, we investigated the role of cancer cells in the autophagy of SCs by upregulating ATG7 expression, which is mediated by neurotrophic factors, such as NGF, secreted by cancer cells. The autophagy of SCs can promote the outgrowth of nerve axons, which can act as a bridge in the occurrence and development of perineural invasion in PanCa.

## Methods

### Reagents and antibodies

Cytokines, antibodies and reagents were purchased from the indicated suppliers: nerve growth factor (NGF; Peprotech, 450–01), RO 08–2750 (RO; NGF inhibitor, GLPBIO, Cas No. 37854–59-4), chloroquine diphosphate salt (CQ; lysosomal inhibitor, Sigma–Aldrich, C6628), hematoxylin and eosin (HE, Beyotime Biotechnology, C0105), and 4′,6-diamidino-2-phenylindole (DAPI, Beyotime Biotechnology, C1002).

The following primary antibodies were used for immunofluorescence staining and western blotting: anti-cleaved caspase-3 (rabbit, Abcam, ab32042), anti-P62 (rabbit, Abcam, ab109012), anti-LC3 (rabbit, Sigma-Aldrich, ABC929), anti-GFAP antibody (rabbit, Abcam, ab68428), anti-GFAP (mouse, Cell Signaling Technology, 3670 T) anti-NF-09 (mouse, Abcam, ab7794), anti-NF-H (mouse, Santa Cruz, sc-133,165), anti-ATG7 (rabbit, Bioworld, BS6046), anti-GAPDH (mouse, Proteintech, 60,004–1-Ig), and anti-ATG5 (rabbit, Abcam, ab108327). The secondary antibodies were as follows: goat anti-mouse IgG (H + L) HRP (SparkJade, EF0001) and goat anti-rabbit IgG (H + L) HRP (SparkJade, EF0002).

### Cell lines and cell culture

The human PDAC cell lines PANC-1 and BxPC-3 were purchased from the Chinese Academy of Sciences Cell Bank of Type Culture Collection (CBTCCCAS, Shanghai, China). PANC-1 and BxPC-3 cells were cultured in Dulbecco’s modified Eagle’s medium (DMEM; Gibco; Thermo Fisher Scientific, USA) or Roswell Park Memorial Institute 1640 (RPMI 1640; Gibco; Thermo Fisher Scientific, USA) with 10% fetal bovine serum (FBS; Biological Industries) at 37 °C with 5% CO^2^. Rat RSC96 Schwann cells (SCs) were obtained from the Chinese Academy of Sciences Cell Bank of Type Culture Collection and cultured in 5% CO^2^ at 37 °C in Dulbecco’s modified Eagle’s medium (DMEM; Gibco; Thermo Fisher Scientific, USA) containing 10% fetal bovine serum (FBS; Biological Industries) and 1% penicillin/streptomycin (Gibco; Thermo Fisher Scientific, USA).

### Coculture of DRG or SCs with cancer cells

Newborn rats were obtained from the laboratory animal center of Xi’an Jiaotong University. As previously described, DRGs were isolated from newborn rats and then washed in cold PBS. After DRGs were embedded in 10 μl of Matrigel (Corning; REF 354234) in a 24-well plate (LabServ; 310,109,007), they were cultured in 5% CO^2^ at 37 °C in DMEM containing 10% FBS. Coculture of cancer cell DRGs or cancer cell SCs was performed as previously described with modifications [17]. On Day 1, a droplet of PANC-1 or BxPC-3 cells (the cell suspension was quantified and adjusted to approximately 2 million cells/mL) was added to a 24-well plate and cultured normally for attachment. On Day 2, a drop of Matrigel (in which a DRG will be plated) or a drop of SCs (2 million cells/mL) was seeded beside the drop of cancer cells at a distance of 1 mm (distance controlled under a microscope with a scale). The recruitment of neurons and SCs by cancer cells was monitored over 3 continuous days.

### Western blotting analysis

Cell lysates were extracted from cultured cells with RIPA buffer (50 mM Tris, pH 8.0, 150 mM NaCl, 0.1% SDS, 1% NP40 and 0.5% sodium deoxycholate) containing proteinase inhibitors (1% inhibitor cocktail and 1 mM PMSF) (Roche Applied Science, Germany) by incubation for 10 min on ice and centrifugation at 12000 × g for 15 min at 4 °C. The concentration of the protein lysates was measured using the BCA Protein Assay Kit (Beyotime Biotechnology, P0012). Eighty micrograms of protein was separated by sodium dodecyl sulfate-polyacrylamide gel electrophoresis (SDS-PAGE, 8–12%) and transferred onto polyvinylidene fluoride membranes (PVDF, Merck Millipore, Billerica, MA, USA). After being blocked in 5% bovine serum albumin for 2 h, the membranes were incubated with primary antibody at 4 °C overnight. The primary antibodies were diluted in 5% bovine serum albumin as follows: LC3 (1:1000), P62 (1:1000), ATG-5 (1:1000), ATG-7 (1:1000), NGF (1:1000), cleaved PARP (1:500), cleaved caspase-3 (1:1000) and GAPDH (1:500). The next day, the membranes were washed with PBST buffer for 5 min 3 times and then incubated with peroxidase-conjugated secondary antibodies for 1 h at room temperature. After the membranes were washed with PBST buffer for 5 min 3 times again, they were visualized with an ECL chemiluminescent detection system (Bio-Rad, USA). Immunoreactive bands were visualized using the ChemiDoc TM XRS + Imaging System (Bio-Rad, 1,708,195). Densitometric quantification of the membranes was performed using ImageJ software (NIMH, National Institutes of Health, USA). The experiments were repeated three times.

### Double immunofluorescence assay

Cells and DRGs were plated on 24-well chamber slides and allowed to attach overnight. Following drug treatment, the cells and DRGs were fixed in 4% paraformaldehyde for 30 min and then washed with PBS 3 times. Next, the samples were blocked in 5% bovine serum albumin (Sigma-Aldrich, Germany) for 1 h and then incubated with the primary antibody overnight. The samples were washed with PBS 3 times, and corresponding fluorescent secondary antibodies (Alexa Fluor 488 and 594, 1:2000, Thermo Fisher Scientific) were added for 1 h at room temperature in the dark. Nuclei were stained with DAPI (1:5000) for 15 min in the dark. Cells and DRGs were imaged using laser scanning confocal microscopy (Nikon A1R/A1).

### Cell proliferation assay

The cell proliferation rate was measured by MTT assays. The cells were seeded in 96-well plates at a density of 0.5*10^4^ PANC-1 cells and 1 * 10^4^ BxPC-3 and RSC96 cells per well and incubated overnight in medium containing 10% FBS. The DMSO (VETEC, Sigma-Aldrich) concentration was adjusted to 0.4%. The cells incubated in serum-free medium were used as the control group. Following incubation for 24, 48 and 72 h at 37 °C, 20 μL of MTT reagent (Sigma-Aldrich; USA) was added to each well, and the cells were incubated for 4 h at 37 °C with 5% CO^2^. Subsequently, the medium was removed completely, and 150 μL of DMSO was added to each well at 37 °C. After oscillation for 15 min, the optical density (OD) value was measured by a microplate autoreader (Bio-Tek Instruments, Winooski, USA) at 490 nm. The relative proliferation rate was characterized as (OD (intervention group)-OD (blank))/(OD (control group)-OD(blank)).

### Cell migration and invasion assays

A Transwell chamber (pore size, 8.0 μm; Millipore, Billerica, USA) with Matrigel (for invasion assays) coating was inserted into a 24-well culture plate. For the invasion assay, 8 μm pore inserts were coated with Matrigel diluted in DMEM (Matrigel:DMEM = 1:8). NGF+/−PANC-1, BxPC-3 and ATG7+/− RSC96 cells were cultured in 6-well plates in medium containing 1% FBS for 24 h before treatment. PCa cells (200 μL, cell density adjusted to 1 × 10^6^ cells/mL) with or without different conditioned media (CM) were seeded in the top chamber with 1% FBS, and 500 μl of culture medium containing 20% FBS was added to the lower chamber as a chemoattractant. The Transwell chamber was incubated for 24 h. The invaded cells on the bottom surface of the filter were fixed in methanol and stained with 1% crystal violet solution (Beyotime Technology; C0121) for 15 min, while the uninvaded cells on the upper chamber were removed by a cotton swab. Cell migration and invasion were determined by counting the stained cells in 10 randomly selected fields under a light microscope.

### Wound healing assay

Pancreatic cancer cells and Schwann cells were grown to confluence in 6-well plates. The monolayer was then artificially wounded using a sterile 200-μl pipette tip. Cell debris was removed by washing the monolayer with PBS. The cells were then incubated with different CMs or drug interventions. Wound closure was monitored by photographing cell migration into the wound at various time points at the same spot with an inverted microscope equipped with a digital camera. The extent of healing was dependent on the ratio of the difference between the original and the remaining wound areas compared with the original wound area.

### Live imaging of cocultured cancer cells

Dynamic interactions between DRG and pancreatic cancer cells with or without conditioned medium (CM) were tested in a Matrigel-based 3D culture system. Images were recorded every 24 h for 72 h after treatment with different drugs. Directionality and density analyses of nerve fibers grown from DGR were performed with ImageJ using Manual Tracking. Quantification of growth clusters is shown as the number of clusters indicating the migration of cancer cells.

### Transfection and lentivirus infection

Cells were transfected with plasmid DNA using Lipofectamine 2000 and shRNA using Lipofectamine RNAiMAX transfection reagent (both from Thermo Fisher Scientific) following the manufacturer’s protocol. Virus packaging was performed in 293 T cells after cotransfection of plasmid with the packaging plasmid psPAX2 and envelope plasmid pMD2.G using Lipofectamine 3000. Viruses were harvested 48 h after transfection, and viral titers were determined. Target cells were infected with recombinant lentivirus-transducing units in the presence of 8 μg/ml polybrene (Sigma-Aldrich).

### Knockdown of NGF and ATG7 expression by lentiviral shRNA

Pancreatic cancer and RSC96 cells were infected with retrovirus containing the recombinant lentiviral vector with NGF (GTCCATGTTGTTCTACACTCT) and ATG7 (GCACAACACCAACACACTTGA) shRNA, respectively. Cells were cultured with 2 μg/mL puromycin (Beyotime Technology; ST551) for 4 weeks, and puromycin-resistant clones were selected. Knockdown of NGF and ATG7 was detected by measuring NGF and ATG7 protein levels by western blot analysis with anti-NGF and anti-ATG7 antibodies.

### Enzyme-linked immunosorbent assay (ELISA)

Conditioned medium obtained from different cells of the culture system was collected at 24, 48 and 72 h after the culture, centrifuged (1200 rpm) for 10 min and frozen at −80 °C until analyses. The levels of NGF were tested by an enzyme-linked immunosorbent assay (ELISA) (Multi Sciences; EK1141–96) according to the manufacturer’s instructions.

### Murine sciatic nerve injection

Female athymic BALB/c nu/nu mice approximately 4–6 weeks old were obtained from the laboratory animal center of Xi’an Jiaotong University. All animal experiments were performed according to approved Institutional Animal Care and Use Committee (IACUC) protocols. Nude athymic mice were anesthetized using isoflurane (1–3%), and their sciatic nerves were exposed as previously described with modification. A sciatic nerve tumor model of pancreatic cancer was established by implanting 0.5 × 10^6^/50 μl PANC-1 and BxPC-3 cells in a PBS + Matrigel mixture. After injection beside the right sciatic nerve, the incision was sutured aseptically. Tumor size and body weight were measured every 3 days. Totally 24 mice were given the tumor cell injection (12 mice received PANC-1 and 12 mice received BxPC-3 injection). The monitoring on mice and the animal welfare were properly taken during the whole experiment. Once the tumor became palpable (approximately 1 week after injection), the mice were randomized into 4 groups (3 mice in each group,) for drug intervention. The mice were intraperitoneally injected with RO (13.75 mg/kg), chloroquine (10 mg/kg [18]), a combination of the two drugs, or vehicle twice per week. Tumor growth (longest diameter and shortest diameter) and body weight (mg) were measured every week for 28 days. No animal losses during the experiment.

### Measurement of sciatic nerve function

Sciatic nerve function was measured every 3 days as described previously. Two measurement scores employed to assess the influence of cancer cell xenografts on sciatic nerve function are as follows:

- Sciatic function index (SFI): calculated as the spread length (mm) between the first and fifth toes of the mouse hind limbs.
- Limb function: graded according to the hind limb paw response to manual extension of the body, from 4 (normal) to 1 (total paw paralysis).

### Transmission electron microscopy

For transmission electron microscopy (TEM) examination, 2 × 10^6^ cells centrifuged as cell bulk or 1 mm3 of tissue sample from sciatic nerve was fixed in a mixture of 3% glutaraldehyde at 4 °C for 2 h. Then, the samples were gently washed with PBS 3 times and fixed in 1% osmium tetroxide for 2 h at room temperature. After three washes with PBS 3, fixed specimens were dehydrated through a graded series of ethanol solutions and then embedded in acetone and Epon 812 resin (1:1) for 30 min. After additional infiltration in Epon 812 for 2 h, the samples were cut into ultrathin sections, placed on 200-mesh copper grids and stained with uranium acetate and lead nitrate for 30 min. Sections were observed with a Hitachi H-7650 electron microscope at 80 kV (Hitachi, Tokyo, Japan).

### Statistics

Unless otherwise indicated, all results are presented as the mean ± SEM of triplicate experiments, and statistical comparisons between different groups were performed by 2-tailed Student’s t test or 1-way ANOVA with multiple comparisons corrections. For all statistical analyses, differences of *P* < 0.05 were considered statistically significant. GraphPad Prism software version 4.0/7.0 (GraphPad Software) and SPSS 22.0 software were used for data analysis.

## Results

### Schwann cell autophagy is activated in PNI

**Fig. 1:**
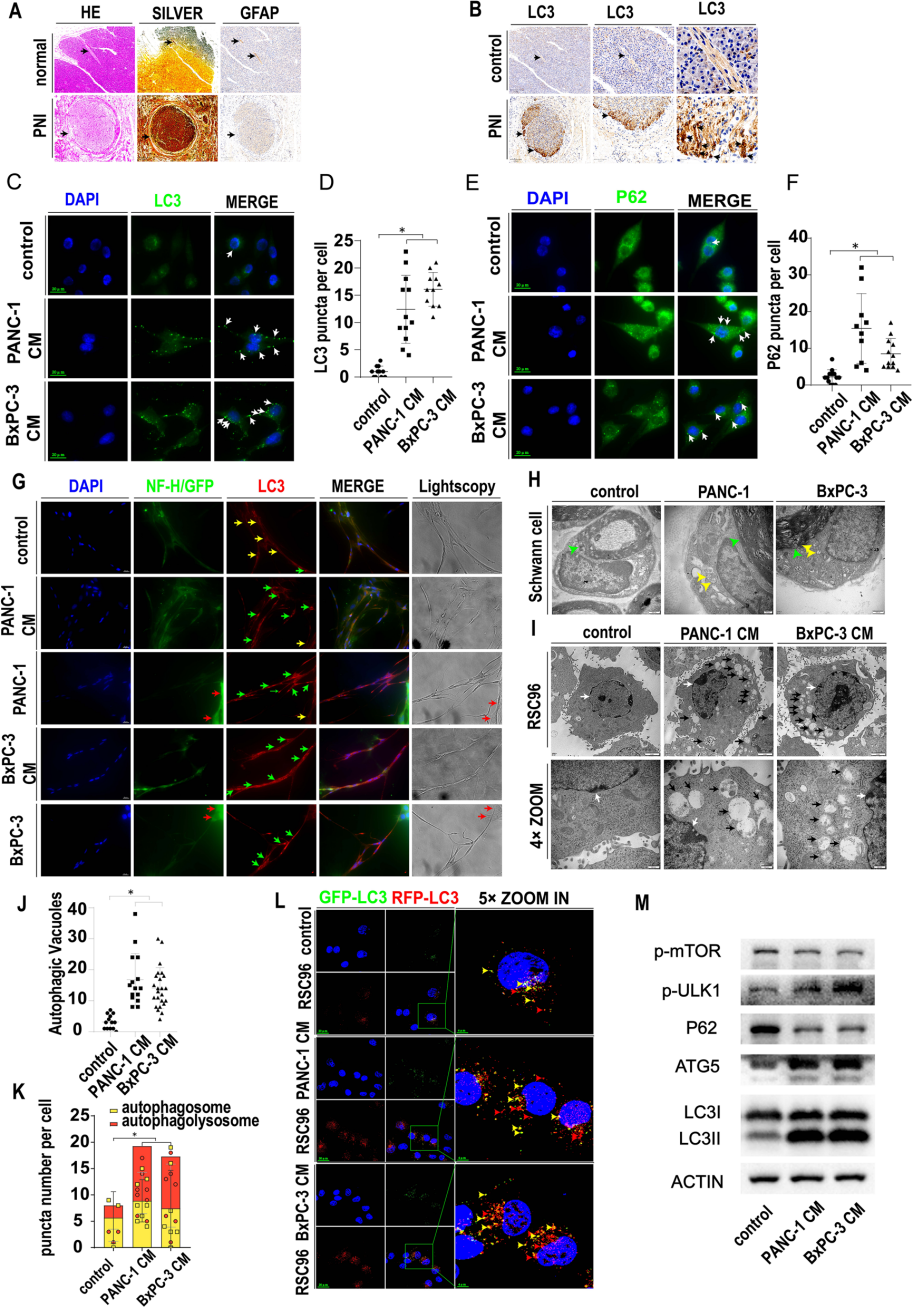
Schwann cell autophagy is activated in PNI. **A** Normal neurons in pancreatic parenchyma and perineural invasion in pancreatic cancer tissue. HE: HE staining showing the general morphology. SILVER: silver staining showing neurons. GFAP: GFAP IHC staining labeling Schwann cells. Black arrows indicate neurons. **B**. Representative LC3 immunohistochemistry in tissue adjacent to carcinoma (control) and PanCa (PNI) tissue, scale bar 200 μm. Black arrows indicate LC3 positive neurons at the same position compared with Fig. 1A. **C**. LC3 immunofluorescence (green) of Schwann cells treated with the control or PanCa-conditioned medium. White arrow: positive LC3 puncta indicating autophagic vacuoles. **D**. Statistics of LC3 puncta number in Schwann cells treated with the control or PanCa-conditioned medium (cell images are in Fig. 1C) (* *p* < 0.05). **E**. P62 immunofluorescence (green) of Schwann cells treated with the control or PanCa-conditioned medium. White arrow: positive P62 puncta indicating autophagic vacuoles. **F**. Statistics of P62 puncta number in Schwann cells treated with the control or PanCa-conditioned medium (cell images are in Fig. 1E) (* *p* < 0.05). **G**. Dorsal root ganglion (DRG) monoculture or coculture with PanCa cells or PanCa CM. DAPI labeled with blue, NF-H labeled with green, LC3 labeled with red. PANC-1 and BxPC-3 cell lines labeled with GFP are shown in green. Light microscopy indicates neurofilament and Schwann cell morphology. Red arrow: PANC-1 or BxPC-3 cells. Yellow arrow: autophagy-inactivated Schwann cells. Green arrow: autophagy-activated Schwann cells. **H**. Representative TEM image of sciatic nerves with or without PDAC cancer cell line injection. Green arrow: myelin sheath formed by Schwann cells. Yellow arrow: autophagosomes in Schwann cells. **I**. Representative TEM image of the RSC96 cell line with or without PanCa cell-conditioned medium. White arrow: cell nucleus. Black arrow: autophagosomes in Schwann cells. **J**. Statistics of autophagic vacuole number in RSC96 cells treated with the control or PanCa-conditioned medium (cell TEM images are in Fig. 1I) (* *p* < 0.05). **K**. Statistics of red and yellow puncta number in RSC96 cells treated with control or PanCa-conditioned medium (cell images are in Fig. 1L) (* *p* < 0.05). **L**. Autophagic flux detection of RSC96 cells treated with the control or PanCa-conditioned medium. LC3 labeled in GFP (green) and RFP (red) and merged as yellow. GFP is unstable in low pH while RFP is stable in low pH. In autophagosome when not fused with lysosomes,the GFP and RFP are both positive to be merged as yellow. When fused with lysosomes,the autophagosomes turn into autophagolysosomes and GFP will be quenched. So the autophagolysosomes are red. Red arrow: autophagolysosomes. Yellow arrow: autophagosome. PanCa-conditioned medium could promote autophagy flux in RSC96 cell line. **M**. Western blotting of RSC96 cells treated with the control or PanCa-conditioned medium. Several autophagy related proteins were detected. Actin was used as a loading control. PanCa-conditioned medium could inhibit p-mTOR while promote p-ULK1 and ATG5 expression in RSC96 cell line. Moreover, PanCa-conditioned medium could promote the conversion of LC3I to LC3II and the degradation of P62

To confirm the existence of Schwann cells and explore their autophagic status in PNI, we selected 10 PanCa tissues and tissues adjacent to carcinoma for our clinical study. Schwann cells were labeled with GFAP, and neurons were stained with silver staining (Fig. 1A). As shown in Fig. 1A, the nerve structure was shown by HE staining. In PanCa tissue with PNI, PanCa cells invade the nerve (arrow) and damage the integrity of the epineurium. However, the nerve epineurium in tissue adjacent to carcinoma (arrow) remained intact. The area of the nerve section was larger in the cancerous tissue than in the tissue adjacent to carcinoma. Silver staining and GFAP immunohistochemical staining confirmed the specific presence of nerves and Schwann cells (arrow). To evaluate the levels of autophagy in the cancerous tissue and the tissue adjacent to carcinoma, we performed LC3 immunohistochemical staining of continuous serial sections of pancreatic parenchyma (Fig. 1B). As shown in Fig. 1B, the pancreatic parenchyma had a relatively low level of positive LC3 staining, indicating a basal level of autophagy in the pancreatic parenchyma. Compared with the normal pancreatic parenchyma adjacent to cancer, the cancerous tissue had a higher LC3 level, indicating that autophagy is activated in pancreatic cancer. Moreover, LC3 expression was even stronger in nerves than in pancreatic cancer tissue and had the same position as GFAP-positive Schwann cells. With the magnification enlarged, we found that the marginal area of the nerve surrounded by cancerous tissue has higher LC3 expression than that in the center of the nerve, indicating that the interaction between the cancer cells and the nerve can promote the autophagy of Schwann cells.

Similarly, in a mouse model of PNI established by injecting cancer cells into the sciatic nerve, Schwann cell autophagy was evaluated by TEM. As shown in Fig. 1H, autophagosomes (yellow arrow) were detected in Schwann cells in the cancer cell injection groups but not in the control groups (myelin is indicated by the green arrow). The green arrow indicates myelin, and the yellow arrow indicates APs in Schwann cells. Moreover, in an in vitro model of PNI constructed by a DRG-PanCa culture system, the autophagic status of Schwann cells was evaluated. Schwann cells were confirmed by IF labeling of GFAP and NF-H (yellow arrow, Fig. S1A). The DRG neurofilament was labeled with NF09 or NF-H, while Schwann cells near the neurofilament were labeled with DAPI (Fig. S1B). The autophagic status was evaluated by LC3 IF labeling (Fig. S1C). The results showed that both direct coculture of DRGs with PanCa cells and indirect coculture of DRGs with PanCa CM could activate autophagy in Schwann cells (Fig. 1G). The yellow arrow indicates autophagy-negative Schwann cells, while the green arrow indicates autophagy-positive Schwann cells. The red arrow indicates PanCa cells. The above results confirmed the existence of autophagy in Schwann cells in perineural invasion, which is activated by interactions with cancer cells.

To determine whether autophagic activation and autophagic flux in SCs in PNI originated from paracrine cancer cells, we used an indirect coculture system of the SC cell line RSC96 and PanCa cell line-derived conditioned medium in our in vitro experiment. As shown in Fig. 1C and Fig. 1D, SCs had low levels of LC3 puncta in the basal state. The LC3 puncta represents the autophagic vacuoles and can reflex the autophagy state of the cells. The accumulation of LC3 puncta in cells may due to the upregulation of the autophagy initiation or the blockade of the autophagy flux. The LC3 puncta number significantly increased with the coculture of PANC-1 (CM1)- and BxPC-3 (CM2)-conditioned medium for 48 h. P62 puncta represents the autophagic vacuoles and can reflex the autophagy degradation state. The P62 puncta accumulation may due to the upregulation of autophagy initiation or the inhibition of autophagy degradation. The P62 puncta number also significantly increased with the coculture of PANC-1 (CM1)- and BxPC-3 (CM2)-conditioned medium for 48 h (Fig. 1E and Fig. 1F). In addition, ATG5 expression was found to increase in the CM group by IF (Fig. S1E).

We next analyzed the morphological structures of autophagy in SCs by transmission electronic microscopy (TEM). Autophagosomes (APs) are distinctly visible under TEM as 2 parallel membrane layers wrapping the substrate. We used this criterion to quantitate the number of APs in our experiments. As shown in Fig. 1I, APs (black arrows) were observed within the SCs in all three groups (nuclei are indicated by white arrows). Interestingly, the average number of APs in the CM1 and CM2 groups increased significantly compared with that in the control groups (Fig. 1J, *p* < 0.05).

To confirm that autophagic flux was active during coculture and that the increase in LC3 and P62 was not due to the blockade of autophagolysosome degradation, we transfected GFP/RFP double-fluorescent LC3 lentivirus into SCs for further experiments. As shown in Fig. 1L, the red LC3 dots (indicating the autophagolysosome) and the yellow LC3 dots (indicating autophagosome not infused with lysosome) were greater in the CM group, and the ratio of the red dot to yellow dot increased (Fig. 1K), indicating a fluent and increased autophagic flux in the cocultured SCs compared with the cells in basal conditions. Autophagy-related proteins were also tested in the 3 groups by western blotting assays. As shown in Fig. 1M, CM1 and CM2 increased the expression of the autophagy-initiating proteins p-mTOR, p-ULK1 and ATG5. The ratio of LC3II to LC3I increased in the CM groups, and P62 expression decreased in the CM1 and CM2 groups. The quantitative WB analysis were shown in Fig. S8A.The above results indicated that pancreatic cancer can activate autophagy by a paracrine mechanism, which is related to PNI in PanCa.

### PanCa-related NGF activates SC autophagy

**Fig. 2:**
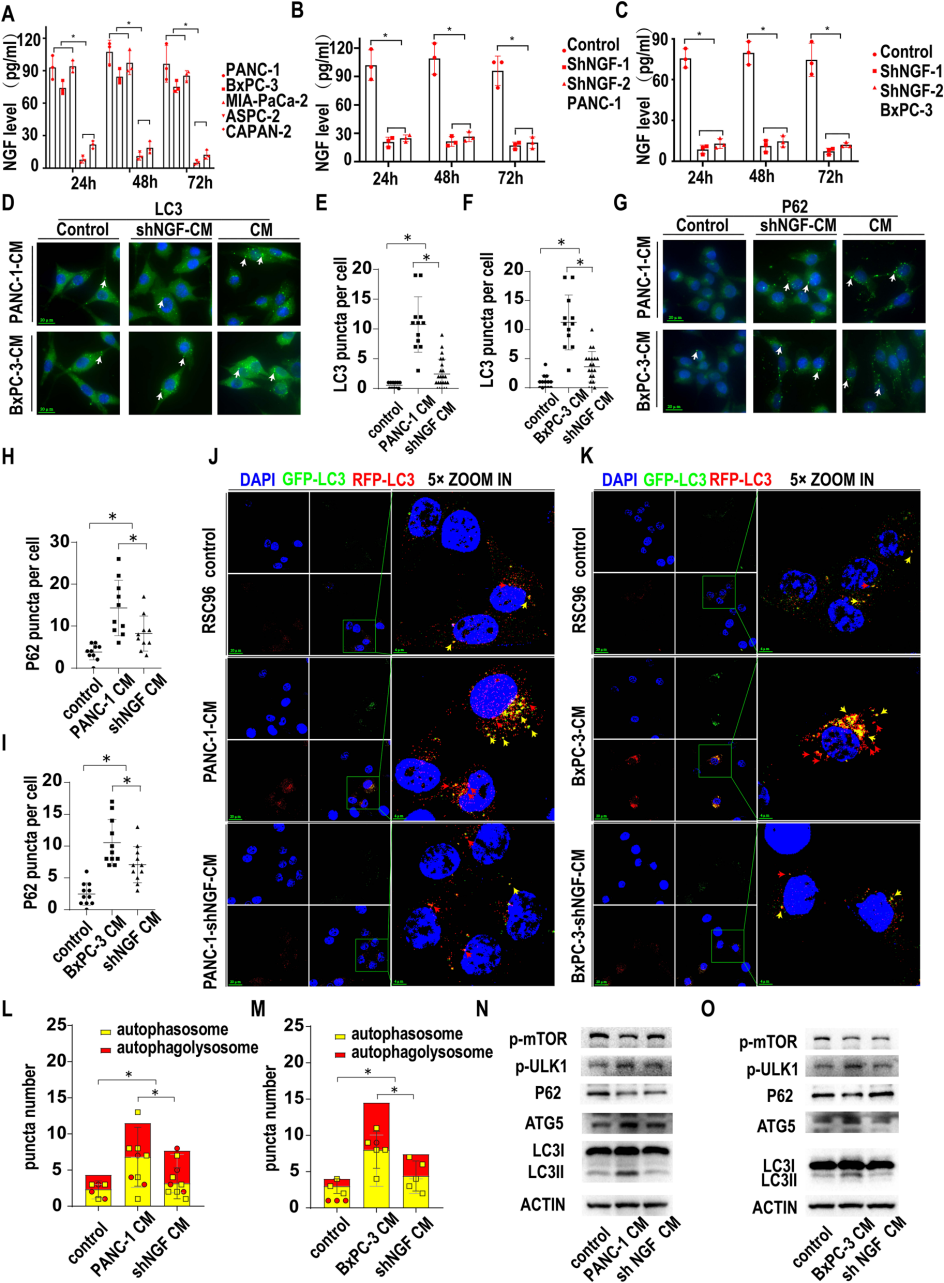
PanCa-related NGF activates SC autophagy. **A**. NGF secretion measured by ELISAs in the serum-free medium of five pancreatic cancer cell lines (* *p* < 0.05). **B**. NGF secretion measured by ELISAs in the serum-free medium of the control and shNGF PANC-1 cancer cell lines (* *p* < 0.05). **C**. NGF secretion measured by ELISAs in the serum-free medium of the control and shNGF BxPC-3 cancer cell lines (* *p* < 0.05). **D**. LC3 immunofluorescence (green) of RSC96 cells treated with the control or shNGF-PanCa CM or PanCa-conditioned medium. White arrows: positive LC3 puncta indicating autophagic vacuoles. **E**. Statistics of LC3 puncta number in RSC96 cells treated with the control or shNGF-PANC-1 CM or normal PANC-1 conditioned medium (* *p* < 0.05). **F**. Statistics of LC3 puncta number in RSC96 cells treated with the control or shNGF-BxPC-3 CM or normal BxPC-3 conditioned medium (* *p* < 0.05). **G**. P62 immunofluorescence (green) of RSC96 cells treated with the control or shNGF-PanCa CM or PanCa-conditioned medium. White arrows:positive P62 punctas indicating autophagic vacuoles. **H**. Statistics of P62 puncta number in RSC96 cells treated with the control or shNGF-PANC-1 CM or normal PANC-1-conditioned medium (* *p* < 0.05). **I**. Statistics of P62 puncta number in RSC96 cells treated with the control or shNGF-BxPC-3 CM or normal BxPC-3-conditioned medium (* *p* < 0.05). **J**. Autophagic flux detection of RSC96 cells treated with the control or shNGF-PANC-1 CM or PANC-1-conditioned medium. LC3 labeled with GFP (green) and RFP (red) and merged as yellow. Red arrow: autophagolysosomes. Yellow arrow: autophagosome. PANC-1-conditioned medium could promote autophagy flux in RSC96 cell line while inhibiting NGF secrection in PANC-1 could partially reverse the effect. **K**. Autophagic flux detection of RSC96 cells treated with the control or shNGF-BxPC-3 CM or BxPC-3-conditioned medium. LC3 labeled with GFP (green) and RFP (red) and merged as yellow. Red arrow: autophagolysosomes. Yellow arrow: autophagosome. PANC-1-conditioned medium could promote autophagy flux in RSC96 cell line while inhibiting NGF secrection in BxPC-3 could partially reverse the effect. **L**. Statistics of red and yellow puncta number in RSC96 cells treated with the control or shNGF-PANC-1 CM or PANC-1-conditioned medium (* *p* < 0.05). **M**. Statistics of red and yellow puncta number in RSC96 cells treated with the control or shNGF-BxPC-3 CM or BxPC-3-conditioned medium (* *p* < 0.05). **N**. Western blotting of NGF in the control or NGF knockdown PANC-1 cell lines. Actin was used as a loading control. Several autophagy related proteins were detected. PANC-1-conditioned medium could inhibit p-mTOR while promote p-ULK1 and ATG5 expression in RSC96 cell line. Moreover, PANC-1-conditioned medium could promote the conversion of LC3I to LC3II and the degradation of P62. Inhibiting NGF secretion of PANC-1 by knocking down NGF could partially reverse the effect. **O**. Western blotting of NGF in the control or NGF knockdown BxPC-3 cell lines. Actin was used as a loading control. Several autophagy related proteins were detected. BxPC-3-conditioned medium could inhibit p-mTOR while promote p-ULK1 and ATG5 expression in RSC96 cell line. Moreover, BxPC-3-conditioned medium could promote the conversion of LC3I to LC3II and the degradation of P62. Inhibiting NGF secretion of BxPC-3 by knocking down NGF could partially reverse the effect

It has been reported that nerve growth factor (NGF) can activate SC autophagy in peripheral nerve injury [16], so we next studied the relationship between PanCa-related NGF and SC autophagy. First, NGF secretion was measured by ELISAs in the serum-free medium of five pancreatic cancer cell lines (Fig. 2A). NGF was present in the medium of all five cancer cell lines. At 24 h, the NGF concentration was 93 pg/ml, 74 pg/ml and 94 pg/ml in PANC-1, BxPC-3 and MIA-PaCa-2 medium, respectively, compared with 7.67 pg/ml and 21.67 pg/ml in ASPC-1 and CAPAN-2 medium, respectively, with significant differences. At 48 h and 72 h, NGF was also detected in the culture medium of all five cancer cell lines at levels comparable to those at 24 h. PANC-1 and BxPC-3 cell lines were selected for the next study due to their higher secretion of NGF.

We stably knocked down NGF expression in PANC-1 and BxPC-3 cells via lentiviral transduction of shRNA targeting the NGF gene. Two clones of shNGF in PANC-1 and BxPC-3 cell lines were selected by puromycin, and the knockdown efficiency was measured by WB of NGF (Fig. S2A). The protein bands were quantified by ImageJ software, and significantly lower NGF expression was found compared with that in the control group (Fig. S2B, C). To explore the secretion of NGF after NGF knockdown, we measured NGF by ELISAs in the serum-free medium of two knockdown cell lines and the parental cells as a control. As shown in Fig. 2B, two single clones of shNGF in PANC-1 cells showed a significantly lower level of NGF secretion than the control cells at 24 h, 48 h and 72 h. Similar NGF expression was also found in the BxPC-3 cell line (Fig. 2C). The ELISA results were consistent with those from western blotting. We chose shNGF-1 knockdown PANC-1 and BxPC-3 cell lines for the next study.

To explore the effect of PanCa cell-secreted NGF on SC autophagy, we cocultured SCs with normal medium (control), PANC-1 and BxPC-3 conditioned medium (CM), or shNGF PANC-1 and BxPC-3 conditioned medium (shNGF-CM). LC3 and P62 expression was tested by immunofluorescence staining. As shown in Fig. 2D, E and F, CM from PANC-1 and BxPC-3 cells significantly increased LC3 puncta in Schwann cells compared with those in the control and shNGF groups. Similarly, CM from PANC-1 and BxPC-3 cells also significantly increased P62 puncta compared with that in the control and shNGF groups (Fig. 2G, H and I). Moreover, the GFP/RFP double labeling LC3 fluorescence assay (Fig. 2J and K) showed that PANC-1 and BxPC-3 CM could increase autophagic flux, while knocking down NGF could partially reverse this effect, indicating that PanCa promotes SC autophagy in an NGF-related manner (Fig. 2L and M). Moreover, we also confirmed that NGF(50 ng/ml,24 h) could promote autophagy in RSC96 cell line by detecting P62 and LC3 puncta (Fig. S2C). And GFP/RFP-LC3 fluorescence assay confirmed that NGF induced P62 and LC3 puncta accumulation was not due to the blockade of autophagy flux (Fig. S2D and Fig. S2E). Autophagy-related proteins were also measured by western blotting. As shown in Fig. 2N, O, higher expression of LC3 II, ATG5 and p-ULK1 was found in the CM group than in the control and shNGF groups, while lower expression of p-mTOR and P62 was found in the CM group than in the control and shNGF groups. The quantitative WB analysis were shown in Fig. S8B and Fig. S8C.The above results indicated that NGF originating from cancer cells partially induced the activation of autophagy in SCs, while other factors may also be involved in SC autophagic induction by cancer cells.

### NGF-induced autophagy promotes the proliferation and migration of SCs

**Fig. 3:**
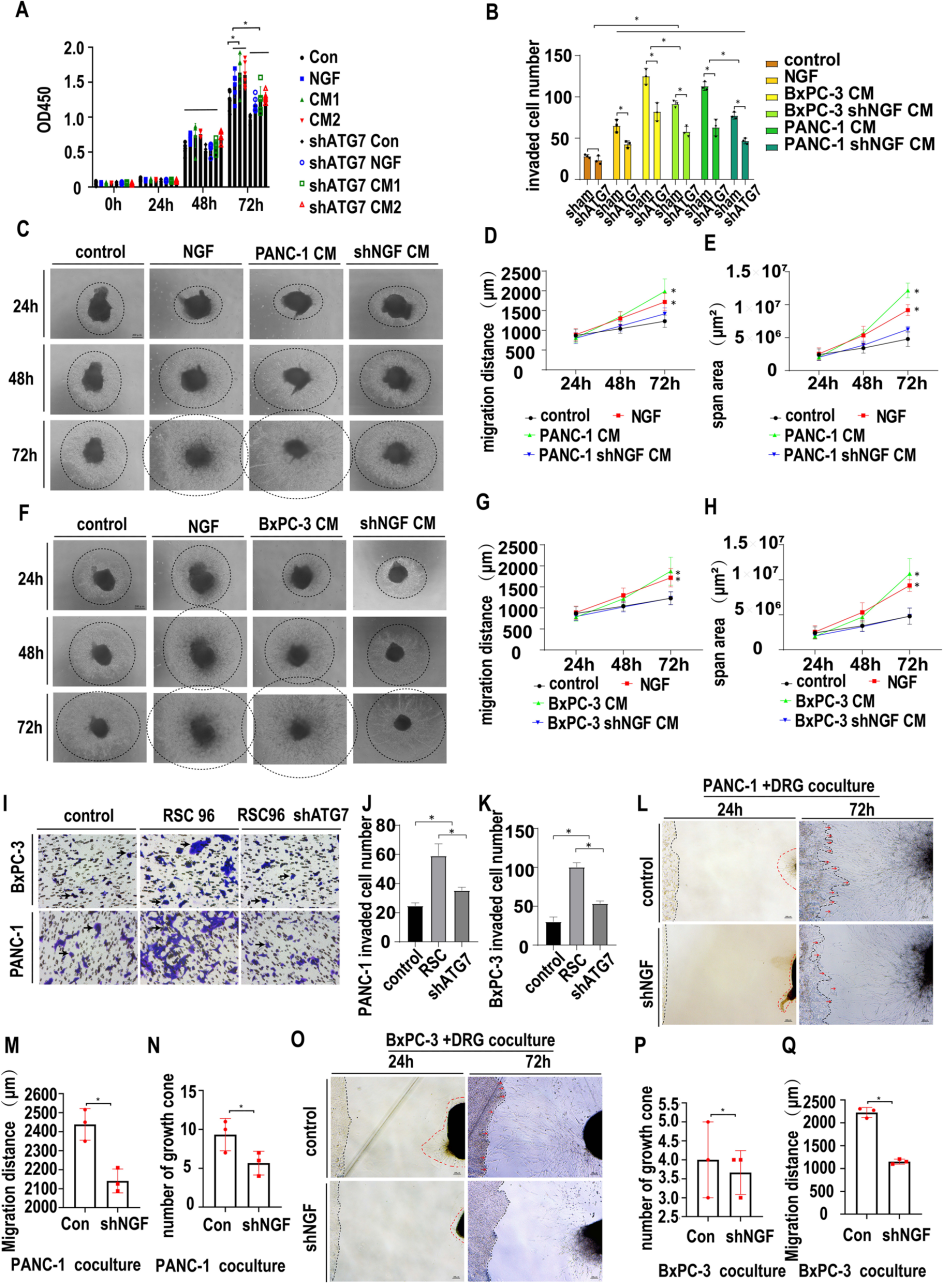
NGF-induced autophagy promotes the proliferation and migration of SCs. **A**. CCK-8 proliferation assays of RSC96 and shATG7-RSC96 cells treated with the control, NGF or PanCa CM. OD 450 nm value was determined at 0 h, 24 h, 48 h and 72 h (* *p* < 0.05).NGF or PanCa CM could promote RSC96 proliferation at 72 h. Knocking down ATG7 on RSC96 could partially reverse the effect. **B**. Transwell migration assays of RSC96 and shATG7-RSC96 cells treated with the control or NGF, PanCa CM or shNGF-PanCa CM (* *p* < 0.05). NGF or PanCa CM could promote RSC96 migration at 24 h. Knocking down ATG7 on RSC96 or knocking down NGF on PanCa could partially reverse the effect. **C**. Dorsal root ganglion monoculture with the control, NGF, PANC-1 CM or shNGF-PANC-1 CM. Light microscopy images were collected at 24 h, 48 h, and 72 h. Dashes indicate the neurofilament scope. **D**. Statistics of the migration distance (μm) of DRG neurofilaments treated with the control, NGF, PANC-1 CM or shNGF-PANC-1 CM (* *p* < 0.05). **E**. Statistics of the migration span (μm^2^) of DRG neurofilaments treated with the control, NGF, PANC-1 CM or shNGF-PANC-1 CM (* *p* < 0.05). **F**. Dorsal root ganglion monoculture with the control, NGF, BxPC-3 CM or shNGF-BxPC-3 CM. Light microscopy images were collected at 24 h, 48 h, and 72 h. Dashes indicate the neurofilament scope. **G**. Statistics of the migration distance (μm) of DRG neurofilaments treated with the control, NGF, BxPC-3 CM or shNGF-BxPC-3 CM (* *p* < 0.05). **H**. Statistics of the migration span (μm^2^) of DRG neurofilaments treated with the control, NGF, BxPC-3 CM or shNGF-BxPC-3 CM (* *p* < 0.05). **I**. Transwell migration assay for BxPC-3 and PANC-1 cells. PANC-1 or BxPC-3 cells were seeded in the upper chamber with 1% serum. No cell, normal RSC96 or shATG7-RSC96 cells in the lower chamber (10% serum were added) as chemoattractants. RSC96 as chemoattractans could promote PanCa cell migration ability while inhibiting RSC96 autophagy by knocking down ATG7 could partially reverse the effect. **J**. Statistics of migrated cells treated with the control, NGF, PANC-1 CM or shNGF-PANC-1 CM (cell image: Fig. 3I, PANC-1) (* *p* < 0.05). **K**. Statistics of migrated cells treated with the control, NGF, BxPC-3 CM or shNGF-BxPC-3 CM (cell image: Fig. 3I, BxPC-3) (* *p* < 0.05). **L**. DRGs were co-cultured with PANC-1 or shNGF-PANC-1 cells. Black dashes indicate the cancer line margin. Red dashes indicate the DRG neurofilament margin. Red arrow: cancer cell growth cone toward DRG. **M**. Statistics of the migration distance (μm) of DRG-derived Schwann cells toward cancer cells (cell image: Fig. 3L) (* *p* < 0.05). **N**. Statistics of growth cone numbers of PANC-1 cells toward DRG (cell image: Fig. 3L) (* *p* < 0.05). **O**. DRGs were co-cultured with BxPC-3 or shNGF-BxPC-3 cells. Black dashes indicates the cancer line margin. Red dashes indicates the DRG neurofilament margin. Red arrow: cancer cell growth cone toward the DRG. **P**. Statistics of growth cone numbers of BxPC-3 cells toward the DRG (cell image: Fig. 3O) (* *p* < 0.05). **Q**. Statistics of the migration distance (μm) of DRG-derived Schwann cells toward cancer cells (cell image: Fig. 3O) (* *p* < 0.05)

ATG7 is essential for autophagic induction. To explore the effect of autophagy on SC biological behavior, we stably knocked down ATG7 in SCs via lentiviral transduction of shRNA targeting the ATG7 gene. Two single clones of shATG7 in SCs with high knockdown efficiency confirmed by WB were selected by puromycin and used for further detection (Fig. S3A). The protein bands were quantified by ImageJ software, and the lower ATG7 expression in the shATG7 group was significant compared with that in the control group (Fig. S3B). To explore the effect of autophagy on SC biological activity, we cultured SCs and shATG7 SCs with normal medium, NGF (10 ng/mL), PANC-1 CM (CM1) and BxPC-3 CM (CM2). The proliferation of SCs and shATG7 SCs was measured by CCK-8 assays at 24 h, 48 h and 72 h. As shown in Fig. 3A, SCs and shATG7 SCs showed no difference in proliferation at 24 h and 48 h, while shATG7 significantly inhibited SC proliferation at 72 h. Moreover, NGF and CM promoted the proliferation of SCs at 72 h, but this proliferation was inhibited by the knockdown of ATG7, indicating that NGF and CM promote SC proliferation in an autophagy-related manner. The migration of SCs and shATG7 SCs was also measured by wound healing assays at 24 h, 48 h, 72 h and 96 h. As shown in Fig. S3C and S3D, the migratory distance showed no difference in all the groups, indicating that the migration of SCs was not changed in a two-dimensional environment. However, the migration of SCs in a 3D environment can be affected by the autophagic state, as shown by Transwell migration assays. As shown in Fig. 3B and Fig. S3E, SCs could be chemoattracted by NGF, CM1 and CM2 when autophagy was active, and the chemoattraction of CM was partially mediated through NGF. However, when SC autophagy was blocked by shATG7, the chemoattraction of NGF, CM1 and CM2 to SCs was significantly attenuated, indicating that NGF, CM1 and CM2 can promote the migration of SCs in a 3D environment in an autophagy-related manner.

To further investigate the role of NGF-induced SC autophagy in PNI, we used an indirect coculture system of dorsal root ganglions (DRGs) and PanCa cell-conditioned medium. To explore the effect of NGF on SC migration, we cultured DRGs with normal medium, NGF (10 ng/mL), PanCa CM (CM) and shNGF PanCa CM (shNGF-CM). The migratory distance of SCs was measured at 24 h, 48 h and 72 h. As shown in Fig. 3C, D and E, NGF and PANC-1 CM promoted SC migration, while shNGF partially abolished the migration-promoting effect of PANC-1 CM. Similar results were obtained from the BxPC-3 CM coculture group (Fig. 3F, G, H). These data revealed that secretion from PanCa cells can promote SC migration partially through NGF.

To examine the effect of the autophagic state of SCs on the migration of PanCa cells, we seeded control SCs and shATG7 SCs in the lower chamber as a chemoattractant to PanCa cells in a Transwell coculture system. After 24 h of coculture, the invaded cells were counted. As shown in Fig. 3I, J and K, SCs chemoattracted PanCa cells, while inhibition of SC autophagy by shATG7 partially reversed the chemoattractive effect. These results indicate that SCs could chemoattract PanCa cells in an autophagy-related manner.

Next, a direct coculture system of DRG and PanCa cell lines was utilized to explore the effect of NGF on PNI. DRGs were cocultured with PANC-1 or shNGF PANC-1 cell lines and monitored under a microscope every day. The number of growth cones of cancer cells and the migratory distance of SCs were measured at 72 h. As shown in Fig. 3L, the number of growth cones and the migratory distance of SCs increased from 24 h to 72 h. The mean number of growth cones was 9.33 in the PANC-1 group compared with 5 in the shNGF PANC-1 group, with a significant difference (Fig. 3N, *p* = 0.03). The mean migratory distance of SCs was 2439 μm in the PANC-1 group compared with 2141 μm in the shNGF PANC-1 group (Fig. 3M, *p* = 0.008). As shown in Fig. 3O, the number of growth cones of cancer cells and the migratory distance of SCs increased from 24 h to 72 h for BxPC-3 cells. The mean number of growth cones was 4 in the BxPC-3 group compared with 3.667 in the shNGF BxPC-3 group, without a significant difference (Fig. 3P, *p* = 0.64). The mean migratory distance of SCs was 2229 μm in the BxPC-3 group compared with 1152 μm in the shNGF BxPC-3 group, with a significant difference (Fig. 3Q, *p* = 0.0001), indicating the essential role of NGF in PNI induction. The above results indicated that NGF, secreted by PanCa cells, can activate autophagy of SCs, promote the proliferation and migration of SCs and then promote PNI in PanCa. Targeting both NGF and autophagy may be a useful treatment method.

### The effect of double targeting of NGF and autophagy on SCs

**Fig. 4:**
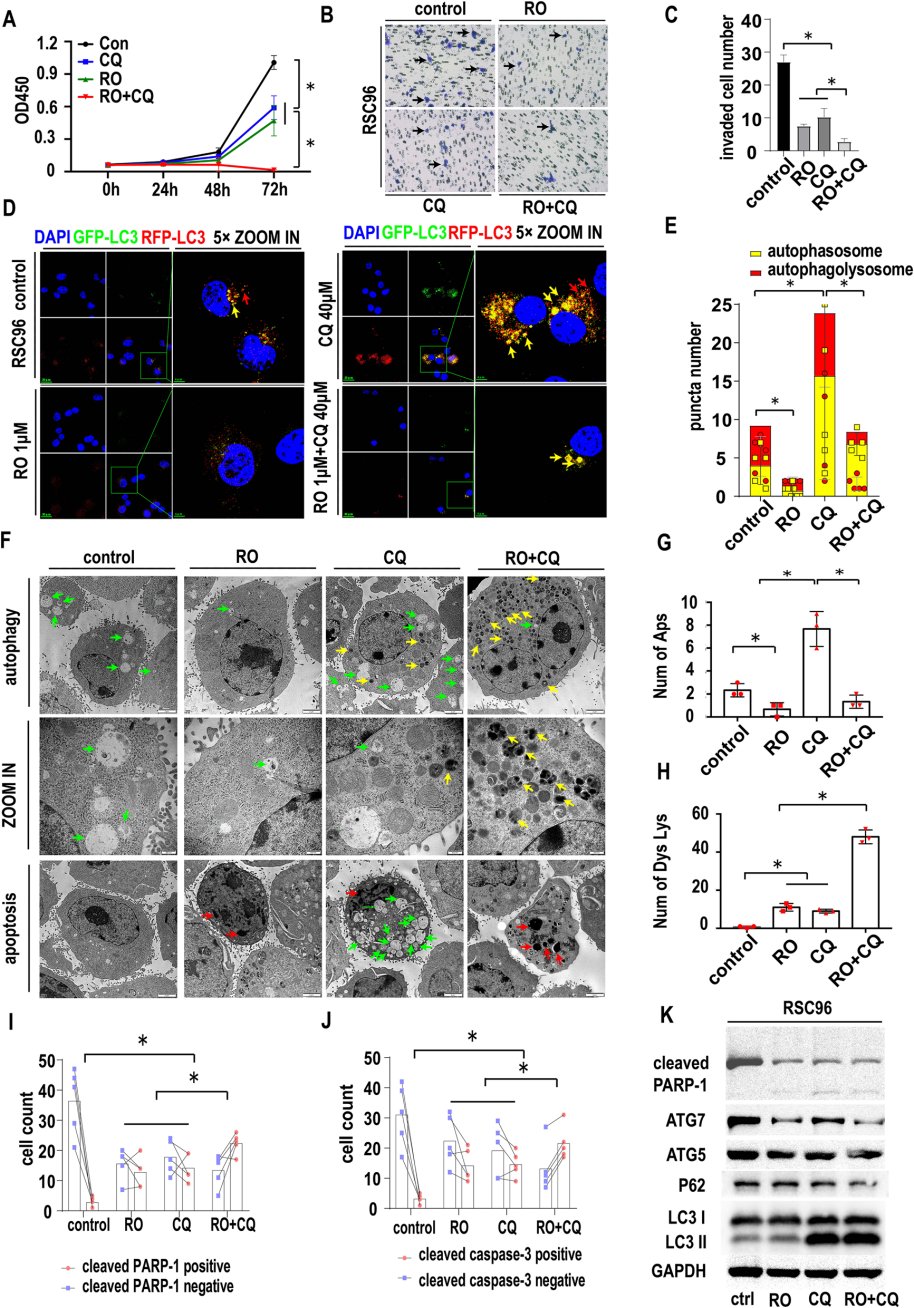
The effect of double targeting of NGF and autophagy on SCs. **A**. CCK-8 proliferation assays of RSC96 cells treated with the control, RO 08–2750 (RO) (1 μM), chloroquine (CQ) (40 μM) or RO + CQ (1 μM + 40 μM). The OD 450 nm value was determined at 0 h, 24 h, 48 h, and 72 h (* *p* < 0.05). **B**. Transwell migration assays of RSC96 cells treated with the control, RO, CQ or RO + CQ.RSC96 were seeded in the upper chamber with 1% serum and the indicated drugs. The lower chamber were 10% serum culture medium. **C**. Statistics of migrated RSC96 cells treated with the control, RO, CQ or RO + CQ (cell image: Fig. 4B) (* *p* < 0.05). **D**. Autophagic flux detection of RSC96 treated with the control, RO, CQ or RO + CQ at 24 h. LC3 labeled with GFP (green) and RFP (red) and merged as yellow. Red arrow: autophagolysosomes. Yellow arrow: autophagosome. RO treatment could reduce both autophagolysosomes and autophagosomes. CQ treatment could increase both autophagolysosomes and autophagosomes. CQ treatment could also increase the ratio of autophagosomes to autophagolysosomes. RO + CQ treatment could increase the ratio of autophagosomes to autophagolysosomes compared with control. RO + CQ treatment could reduce autophagolysosomes and autophagosomes compared with single CQ group. **E**. Statistics of red and yellow puncta number in RSC96 cells treated with the control, RO, CQ or RO + CQ (cell image: Fig. 4D) (* *p* < 0.05). **F**. TEM image of RSC96 cells treated with RO, CQ or RO + CQ at 24 h. Autophagy and apoptosis signs were detected. Green arrow: autophagosome. Red arrow: apoptosis sign. Yellow arrow: dysfunctional lysosome (CQ inhibits the lysosome function and the dysfunctional lysosomes are condensed and shows high density in TEM image). **G**. Statistics of autophagosome numbers of RSC96 cells treated with RO, CQ or RO + CQ (cell image: Fig. 4F) (* *p* < 0.05). **H**. Statistics of dysfunctional lysosome numbers of RSC96 cells treated with RO, CQ or RO + CQ (cell image: Fig. 4F) (* *p* < 0.05). **I**. Statistics of cleaved PARP1-positive and cleaved PARP1-negative RSC96 cell numbers treated with the control, RO, CQ or RO + CQ (cell image: Fig. S4D) (* *p* < 0.05). **J**. Statistics of cleaved caspase-3-positive and cleaved caspase-3-negative RSC96 cell numbers treated with the control, RO, CQ or RO + CQ (cell image: Fig. S4D) (* *p* < 0.05). **K**. Western blotting of RSC96 cells treated with RO, CQ or RO + CQ. GAPDH was used as a loading control. RO, CQ or RO + CQ could induce the expression of cleaved PARP-1. RO, CQ or RO + CQ could inhibit ATG7 expression in RSC96 while could not influence ATG5. RO could inhibit LC3II formation while CQ or RO + CQ could promote LC3II accumulation

To explore the effect of targeting NGF and autophagy on SC biological behavior, we treated SCs with PBS, RO 08–2750 (RO, 1 μM, according to the manufacturers instruction), chloroquine (CQ, 40 μM, according to previous study [19, 20] and our preliminary experiment) and the RO + CQ combination(RO, 1 μM + CQ, 40 μM). The proliferation of SCs was measured by CCK-8 assays at 24 h, 48 h and 72 h. As shown in Fig. 4A, the OD450 value was similar among the 4 groups at 24 h and 48 h without a significant difference. At 72 h, both RO and CQ significantly inhibited the growth of SCs (*p* < 0.05), and the combination of RO + CQ showed even greater inhibition of cell viability. The invasion of SCs was also inhibited by RO, CQ and RO + CQ compared with that of the control group, as shown by Transwell migration assays (Fig. 4B). The number of invaded cells in the RO + CQ group was significantly lower than that of the other three groups (Fig. 4C, *p* < 0.05).

To evaluate the effect of the treatment on the autophagic flux of SCs, we used a GFP/RFP-LC3 IF assay to evaluate autophagic flux. As shown in Fig. 4D and E, after 24 h of treatment, RO inhibited the number of red and yellow dots. CQ treatment could increase the yellow dot number. RO + CQ treatment decreased the red dot number and increased the yellow dot number. These results indicate that RO could inhibit autophagic initiation and that CQ could inhibit autophagic flux by inhibiting the fusion of autophagosomes with lysosomes. Dual targeting of NGF and autophagy by RO + CQ could inhibit autophagic initiation and autophagic flux.

To determine whether the treatment could influence the apoptosis of SCs, we assessed Schwann cell morphology and performed IF for cleaved caspase-3 and cleaved PARP1. The morphology of SCs after treatment is shown in Fig. S4A and statistics of apoptic or non-apoptic cells in Fig. S4B. The green arrows indicate normal SC cells, and the red arrows indicate apoptotic cells. As shown in Fig. S4C, in the basal state, the rate of cleaved PARP1-positive cells (yellow arrow) was low. RO, CQ or RO + CQ increased the rate of cleaved PARP1-positive cells (Fig. 4I). Similar results were obtained by IF of cleaved caspase-3 in SCs (yellow arrow) (Fig. S4D and Fig. 4J).

We next analyzed the morphological structures of SCs under treatment. As shown in Fig. 4F by TEM of Schwann cells, APs were observed within the SCs in all four groups (green arrow). The number of APs (Fig. 4G) was dramatically decreased in the RO group compared with the control group, indicating that RO suppressed the initiation of autophagic flux. It has been proved that CQ could inhibit the function of lysosomes and caused the condensation of lysosomes [19]. Many dysfunctional lysosomes (yellow arrow, condensed and high density in TEM image compared with normal lysosomes) were found in the CQ-treated group, consistent with previous papers showing that CQ inhibits lysosomal function and inhibits the fusion of APs with lysosomes, preventing the degradation of the contents of APs so that autophagic flux cannot be completed (Fig. 4H). Moreover, the number of APs in the CQ group was higher than that in the control group, indicating the accumulation of undegraded APs. However, the number of APs in the RO + CQ group was lower than that in the CQ group, indicating that the initiation of autophagy was inhibited by RO regardless of whether CQ was present. Apoptotic signs were also detected in the RO, CQ and RO + CQ groups, including marginal clusters of chromatin, condensation of the cytosol and formation of apoptotic bodies (red arrow). The above data showed that RO could inhibit NGF-induced autophagic initiation and that CQ could inhibit the degradation of APs. The combination of RO and CQ could inhibit the autophagic process at different levels and inhibit the proliferation and invasion of SCs. Treatment with RO, CQ and RO + CQ could induce Schwann cell apoptosis and might be an effective in preventing autophagy-promoted PNI.

Autophagy- and apoptosis-related proteins were also tested by western blotting. As shown in Fig. 4K, RO treatment inhibited LC3 II expression and increased P62 expression, indicating that RO inhibits autophagic initiation. ATG5 and ATG7 expression was also decreased in the RO group. Cleaved PARP1 expression was slightly increased, indicating that apoptosis was induced in the RO group. CQ treatment increased both LC3 II and P62 expression, indicating that CQ inhibits autophagic flux. ATG5 and ATG7 expression was also decreased in the CQ group. The cleaved PARP1 level was increased in the CQ group. RO + CQ treatment increased LC3II expression, but the level was lower than that after CQ treatment, and P62 expression was decreased, indicating inhibition of autophagic initiation and autophagic flux blockade. The quantitative WB analysis were shown in Fig. S8D.

### The effect of double targeting of NGF and autophagy on cancer cells

**Fig. 5:**
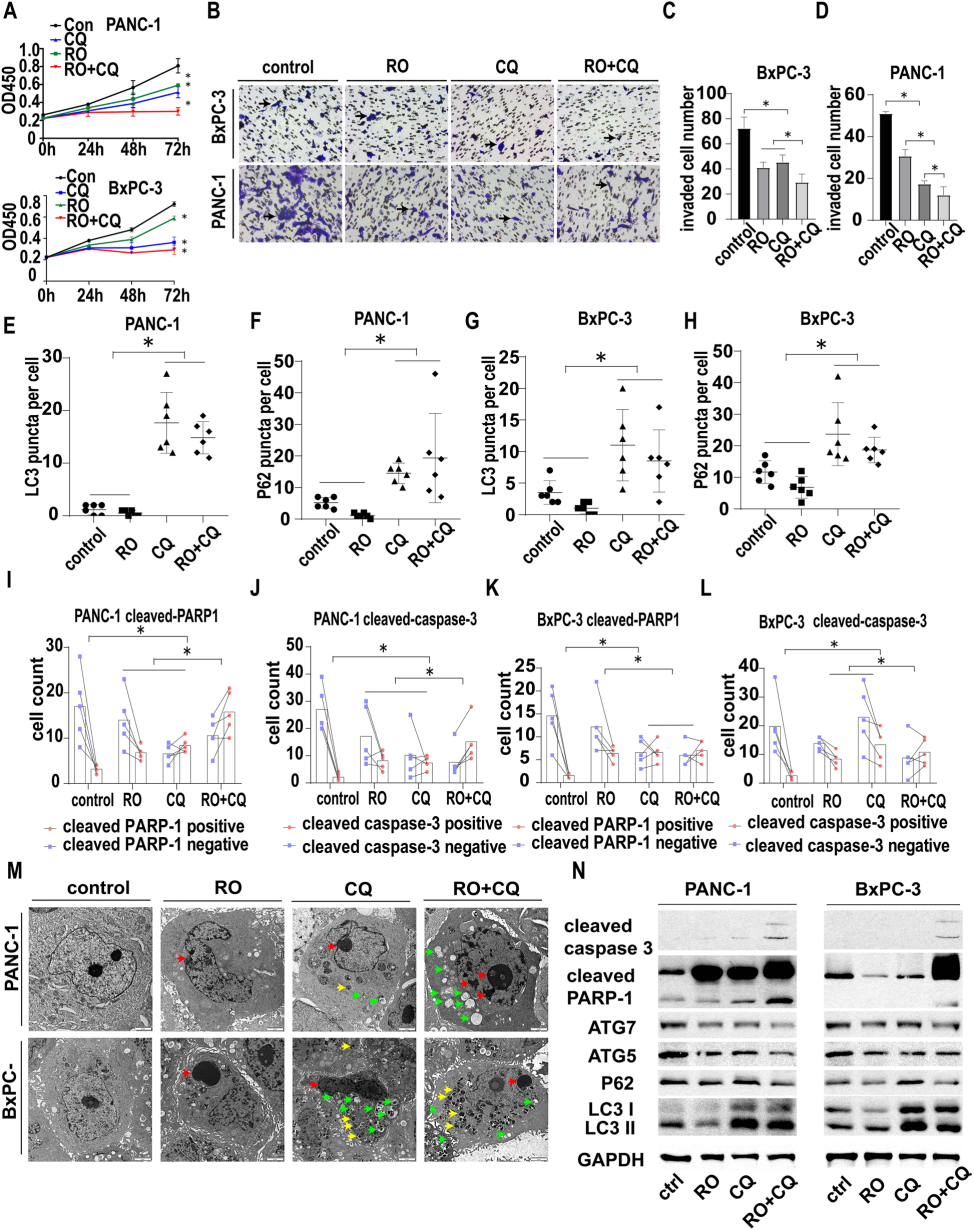
The effect of double targeting of NGF and autophagy on cancer cells. **A**. CCK-8 proliferation assay of PANC-1 and BxPC-3 cells treated with the control, RO (1 μM), CQ (40 μM) or RO + CQ (1 μM + 40 μM). The OD 450 nm value was determined at 0 h, 24 h, 48 h and 72 h (* *p* < 0.05). **B**. Transwell migration assay of PANC-1 and BxPC-3 cells treated with the control, RO (1 μM), CQ (40 μM) or RO + CQ (1 μM + 40 μM) at 24 h. PANC-1 or BxPC-3 were seeded in the upper chamber with 1% serum and the indicated drugs. The lower chamber were 10% serum culture medium. Black arrow: migrated cell. **C**. Statistics of migrated BxPC-3 cells treated with the control, RO (1 μM), CQ (40 μM) or RO + CQ (1 μM + 40 μM) (cell image: Fig. 5B) (* *p* < 0.05). **D**. Statistics of migrated PANC-1 cells treated with the control, RO (1 μM), CQ (40 μM) or RO + CQ (1 μM + 40 μM) (cell image: Fig. 5B) (* *p* < 0.05). **E**. Statistics of LC3 puncta number in PANC-1 cells treated with control, RO (1 μM), CQ (40 μM) or RO + CQ (1 μM + 40 μM) (cell image: Fig. S5A) (* *p* < 0.05). **F**. Statistics of the P62 puncta number in PANC-1 cells treated with control, RO (1 μM), CQ (40 μM) or RO + CQ (1 μM + 40 μM) (cell image: Fig. S5B) (* *p* < 0.05). **G**. Statistics of LC3 puncta number in BxPC-3 cells treated with control, RO (1 μM), CQ (40 μM) or RO + CQ (1 μM + 40 μM) (cell image: Fig. S5C) (* *p* < 0.05). **H**. Statistics of P62 puncta number in BxPC-3 cells treated with control, RO (1 μM), CQ (40 μM) or RO + CQ (1 μM + 40 μM) (cell image: Fig. S5D) (* *p* < 0.05). **I**. Statistics of cleaved PARP1-positive and cleaved PANC-1-negative cell numbers treated with the control, RO (1 μM), CQ (40 μM) or RO + CQ (1 μM + 40 μM) (cell image: Fig. S5E) (* *p* < 0.05). **J**. Statistics of cleaved caspase-3-positive and cleaved caspase-3-negative PANC-1 cell numbers treated with the control, RO (1 μM), CQ (40 μM) or RO + CQ (1 μM + 40 μM) (cell image: Fig. S5F) (* *p* < 0.05). **K**. Statistics of cleaved PARP1-positive and cleaved PARP1-negative BxPC-3 cells treated with the control, RO (1 μM), CQ (40 μM) or RO + CQ (1 μM + 40 μM) (cell image: Fig. S5G) (* *p* < 0.05). **L**. Statistics of cleaved caspase-3-positive and cleaved caspase-3-negative BxPC-3 cell numbers treated with the control, RO (1 μM), CQ (40 μM) or RO + CQ (1 μM + 40 μM) (cell image: Fig. S5H) (* *p* < 0.05). **M**. TEM image of PANC-1 and BxPC-3 cells treated with the control, RO (1 μM), CQ (40 μM) or RO + CQ (1 μM + 40 μM) at 24 h. Autophagy and apoptosis signs were detected. Green arrow: autophagosome. Red arrow: apoptosis sign. Yellow arrow: dysfunctional lysosome. **N**. Western blotting of PANC-1 and BxPC-3 cells treated with the control, RO (1 μM), CQ (40 μM) or RO + CQ (1 μM + 40 μM) at 24 h. GAPDH was used as a loading control

Then, the pancreatic cancer cell lines PANC-1 and BxPC-3 were used to test the effect of the combination of the NGF inhibitor and chloroquine on cancer cells. PANC-1 and BxPC-3 cells were treated with vehicle, RO 08–2750 (RO), chloroquine (CQ) or RO + CQ. As shown in Fig. 5A, the combination of RO + CQ inhibited the growth of PANC-1 cells at 48 h and 72 h compared with that in the other three groups, with a significant difference in CCK-8 assays (*p* < 0.05). Similar results were also observed in BxPC-3 cells at 48 h and 72 h compared with those of the other three groups, with a significant difference in CCK-8 assays.

The invasion of PANC-1 cells was inhibited by RO, CQ and RO + CQ compared with that of the control group, as shown by Transwell migration assays (Fig. 5B). The number of invaded cells in the RO + CQ group was significantly lower than those in the other three groups (Fig. 5C, *p* < 0.05). Similar results were also observed in BxPC-3 cells compared with those of the other three groups, with a significant difference in the Transwell migration assay (Fig. 5D).

To assess the autophagic state of the cancer cells after treatment, we utilized P62 and LC3 IF of PANC-1 and BxPC-3 cell lines, and LC3 puncta and P62 puncta number per cell were counted (Fig. S5A, B, C, D). As shown in Fig. 5E and F, RO treatment did not increase the LC3 and P62 puncta, while CQ and RO + CQ treatment significantly increased the LC3 and P62 puncta. Similar results were obtained in the BxPCc-3 cell line (Fig. 5G, H). These results indicate that targeting NGF in PanCa cells did not influence the autophagic state, while CQ indeed blocked autophagic flux in cancer cells.

To assess the effect of the treatment on the apoptosis of cancer cells, we performed IF for cleaved caspase-3 and cleaved PARP1 (Fig. S5E, F, G, H). As shown in Fig. 5I and J, RO, CQ or RO + CQ treatment activated PARP and caspase-3 and induced apoptosis in the PANC-1 cell line. The rates of cleaved PARP1-positive cells and cleaved caspase-3-positive cells increased after treatment with RO, CQ or RO + CQ. The RO + CQ group exhibited greater apoptosis than the RO or CQ groups. Similar results were obtained in the BxPC-3 cell line (Fig. 5K, L).

We next analyzed the morphological structures of PANC-1 and BxPC-3 cells by light microscopy and TEM. Light microscopy (Fig. S4A) showed that RO, CQ and RO + CQ treatment could change the morphology of PANC-1 and BxPC-3 cell lines, and apoptotic cells are indicated by the red arrow. Apoptosis was also examined in the four groups by TEM (Fig. 5M). We found that the RO, CQ and RO + CQ groups had signs of apoptosis in TEM, including apoptotic bodies and margination of chromosomes (red arrow). Dysfunctional lysosomes (yellow arrow) and APs (green arrow) were also found in the CQ- and RO + CQ-treated groups. Autophagy- and apoptosis-related proteins were also tested in PANC-1 and BxPC-3 cells by western blotting. As shown in Fig. 5N, lower expression of LC3I and LC3II was found in the RO group than in the other groups. The expression of P62, ATG5 and ATG7 was lower in the RO and RO + CQ groups than in the control and CQ groups. We also found that the expression of cleaved PARP1 and cleaved caspase-3 increased in the RO + CQ group, indicating that the combination of the NGF inhibitor RO and the autophagy blocker chloroquine can induce the apoptosis of cancer cells. The quantitative WB analysis were shown in Fig. S8E.The above results indicated that the combination of RO and chloroquine can inhibit autophagic flux and induce apoptosis of cancer cells, resulting in the inhibition of proliferation and invasion.

### The effect of double targeting of NGF and autophagy on PNI

**Fig. 6:**
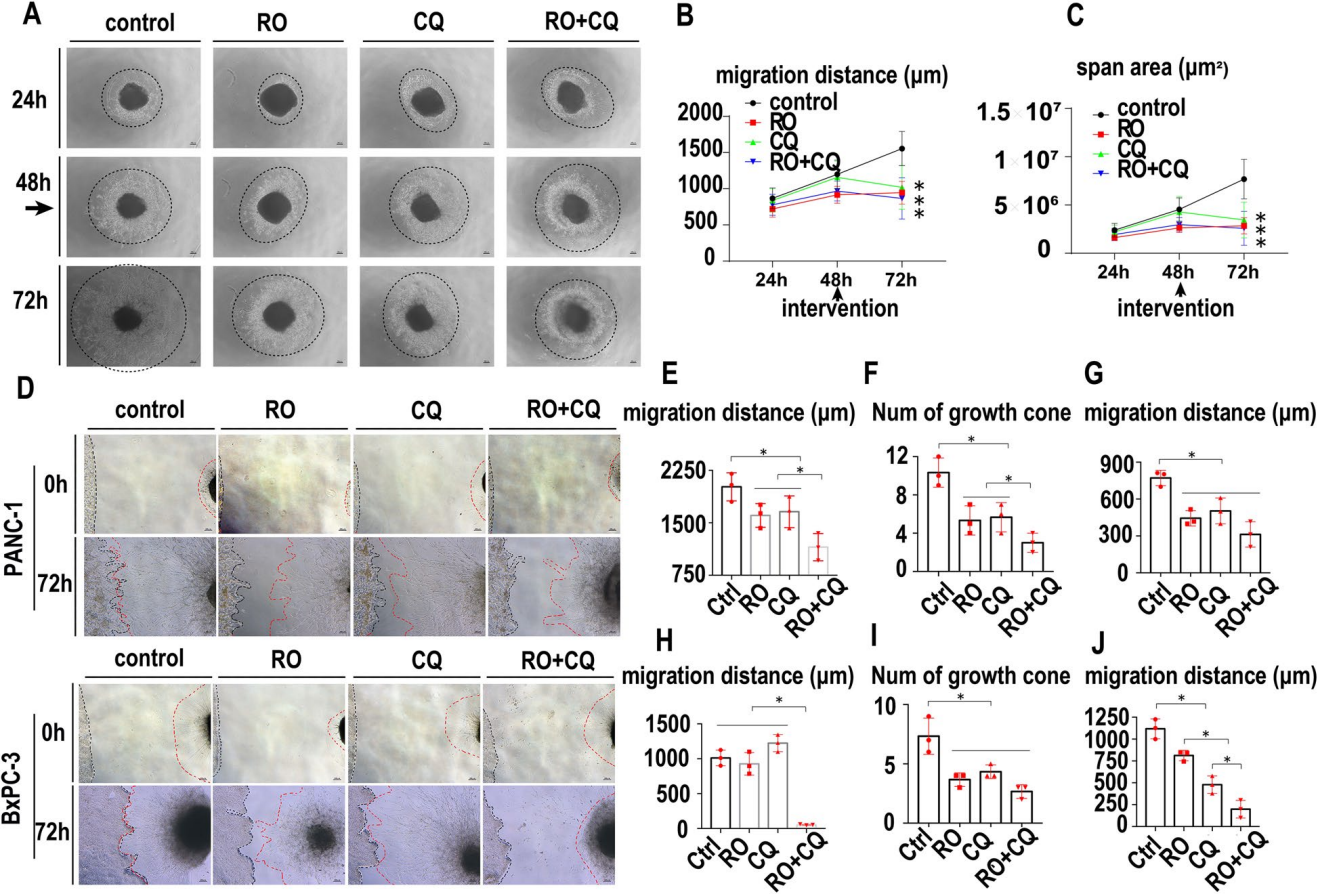
The effect of double targeting of NGF and autophagy on PNI. **A**. Dorsal root ganglion monoculture with the control, RO (1 μM), CQ (40 μM) or RO + CQ (1 μM + 40 μM). The intervention begins at 48 h, and light microscopy images were collected at 24 h, 48 h, and 72 h. Dashes indicate the neurofilament scope. **B**. Statistics of the migration distance (μm) of DRG neurofilaments treated with the control, RO, CQ or RO + CQ (cell image: Fig. 6A) (* *p* < 0.05). **C**. Statistics of the migration span (μm^2^) of DRG neurofilaments treated with the control, RO, CQ or RO + CQ (cell image: Fig. 6A) (* *p* < 0.05). **D**. DRGs cocultured with PANC-1 or BxPCc-3 cells were treated with control, RO (1 μM), CQ (40 μM) or RO + CQ (1 μM + 40 μM). Black dashes indicates the cancer line margin. Red dashes indicates the DRG neurofilament margin. **E**. Statistics of the migration distance (μm) of DRG-derived Schwann cells toward PANC-1 cells treated with the control, RO, CQ or RO + CQ (cell image: Fig. 6D) (* *p* < 0.05). **F**. Statistics of the growth cone numbers of PANC-1 cells toward DRGs treated with the control, RO, CQ or RO + CQ (cell image: Fig. 6D) (* *p* < 0.05). **G**. Statistics of the migration distance (μm) of PANC-1 cells toward DRGs treated with the control, RO, CQ or RO + CQ (cell image: Fig. 6D) (* *p* < 0.05). **H**. Statistics of the migration distance (μm) of DRG-derived Schwann cells toward BxPC-3 cells treated with control, RO (1 μM), CQ (40 μM) or RO + CQ (1 μM + 40 μM) (* *p* < 0.05). **I**. Statistics of growth cone numbers of BxPCc-3 cells toward DRGs treated with the control, RO, CQ or RO + CQ (cell image: Fig. 6D) (* *p* < 0.05). **J**. Statistics of the migration distance (μm) of BxPC-3 cells toward DRGs treated with the control, RO, CQ or RO + CQ (cell image: Fig. 6D) (* *p* < 0.05).K. Statistics of the migration distance (μm) of PANC-1 cells toward DRGs treated with the control, RO, CQ or RO + CQ (cell image: Fig. 6D) (* *p* < 0.05)

To explore the effect of the combination of the NGF inhibitor and chloroquine on PNI, we treated a monoculture of DRGs with the control, RO, CQ and RO + CQ at 48 h after implantation, and the DRG neurofilament outgrowth was evaluated until 72 h post-implantation. As shown in Fig. 6A, the migratory distance of DRG neurofilaments was inhibited significantly by RO, CQ and RO + CQ compared with the control. The average migratory distance was quantified by measuring the mean of different migratory directions of neural outgrowth originating from the edge of the DRG and the outgrowth area (Fig. 6B, C).

We then used NF09 and LC3 double IF to evaluate the autophagic state of the DRG-originated SCs. As shown in Fig. S6A, NF09 labeled neurofilaments, DAPI labeled SC cell nuclei and LC3 labeled autophagy. RO, CQ, and RO + CQ treatment inhibited neurofilament outgrowth. CQ and RO + CQ treatment increased LC3 levels, indicating a blockade of autophagic flux and accumulation of undegraded APs. In the RO treatment group, the ratio of LC3-negative SCs increased compared with that in the control group, thus indicating the inhibition of autophagic initiation. Moreover, the number of migrated SCs from DRGs labeled by DAPI was larger in the control group than in the other groups, especially in the RO + CQ group. The concrete number of SCs was not measured because of the overlay of cells.

To evaluate the effect of the treatment on the apoptotic state of the DRG, we used cleaved caspase-3 IF in the DRG monoculture. As shown in Fig. S6B, the SCs in the control group had a basal level of cleaved caspase-3 labeling defined as cleaved caspase-3 negative. In the RO, CQ and RO + CQ groups, cleaved caspase-3-positive SCs were detected, indicating that RO, CQ and RO + CQ treatment could induce the apoptosis of SCs in the DRG. The green arrow indicates cleaved caspase-3-positive SCs.

We next explored the effect of the combination of an NGF inhibitor and chloroquine on PNI in PanCa. DRGs were cocultured with PANC-1 and BxPC-3 cells. The migratory distance of SCs, the number of growth cones and the migratory distance of cancer cells were measured at 72 h after treatment. The migratory distance of SCs and cancer cells was tested at 0 h of coculture. The growth cone of cancer cells was not found at 0 h of coculture. With the elongation of coculture time, the cancer cells migrated to DRGs with growth cones, and the SCs from DRGs also migrated to cancer cells and touched each other in both coculture models (Fig. 6D, H). The mean migratory distances of SCs were 2014 μm, 1605 μm, 1658 μm and 1152 μm in the control, RO, CQ and RO + CQ groups at 72 h after treatment of the PANC-1-DRG coculture models, respectively. The combination of RO + CQ significantly inhibited the migration of SCs compared with that of the other groups (Fig. 6E, *p* < 0.05). The mean number of growth cones in PANC-1 cells was 10.33, 5.33, 5.66 and 3.00 in the control, RO, CQ and RO + CQ groups at 72 h after treatment, respectively (Fig. 6F). The mean migratory distances of PANC-1 cells were 771.3 μm, 444.3 μm, 503.3 μm and 312.7 μm in the control, RO, CQ and RO + CQ groups at 72 h after treatment, respectively. The single use of RO and CQ and the combination of RO + CQ significantly inhibited the formation of growth cones and migratory distance of PANC-1 cells compared with those of the control group (Fig. 6F, G, *p* < 0.05). The mean migratory distances of SCs were 1012 μm, 927 μm, 1225 μm and 52.33 μm in the control, RO, CQ and RO + CQ groups 72 h after treatment in the BxPC-3-DRG coculture models, respectively. The combination of RO + CQ significantly inhibited the migration of SCs compared with that of the other groups (Fig. 6H, *p* < 0.05). The mean number of growth cones in the BxPC-3 group was 7.33, 3.67, 4.33 and 2.67 at 72 h after treatment in the control, RO, CQ and RO + CQ groups, respectively (Fig. 6I). The mean migratory distances of BxPC-3 cells were 1117 μm, 811.7 μm, 479 μm and 198.3 μm in the control, RO, CQ and RO + CQ groups 72 h after treatment, respectively. The single use of RO and CQ and the combination of RO + CQ significantly inhibited the formation of growth cones and the migratory distance of BxPC-3 cells compared with those of the control group (Fig. 6J, *p* < 0.05).

To evaluate the effect of dual targeting of NGF and autophagy on PNI, we used a PanCa cell and DRG coculture model. After treatment with RO, CQ or RO + CQ, the coculture system was harvested, and NF09 plus cleaved caspase-3 or LC3 double IF was performed. As shown in Fig. S6C and D, applying RO, CQ or RO + CQ increased the cleaved caspase-3-positive cancer cell ratio. The combination of RO and CQ could induce more cancer cells to undergo apoptosis than either RO or CQ alone. SCs originating from the DRG also showed cleaved caspase-3 positivity in the RO, CQ or RO + CQ group compared with the control group. As shown in Fig. S6E and S6F, the use of RO inhibited the LC3 level in DRG-SCs and cancer cells. CQ could block autophagy, thus increasing the LC3 level in cancer cells and DRG-SCs, while the addition of RO with CQ partially reversed the LC3 level. These results indicate that RO could inhibit SC autophagic initiation, while CQ could block autophagic flux. The combined use of RO and CQ could induce SC and cancer cell apoptosis and inhibit PNI in an in vitro model.

### Double targeting of NGF and autophagy inhibits PNI in vivo

**Fig. 7:**
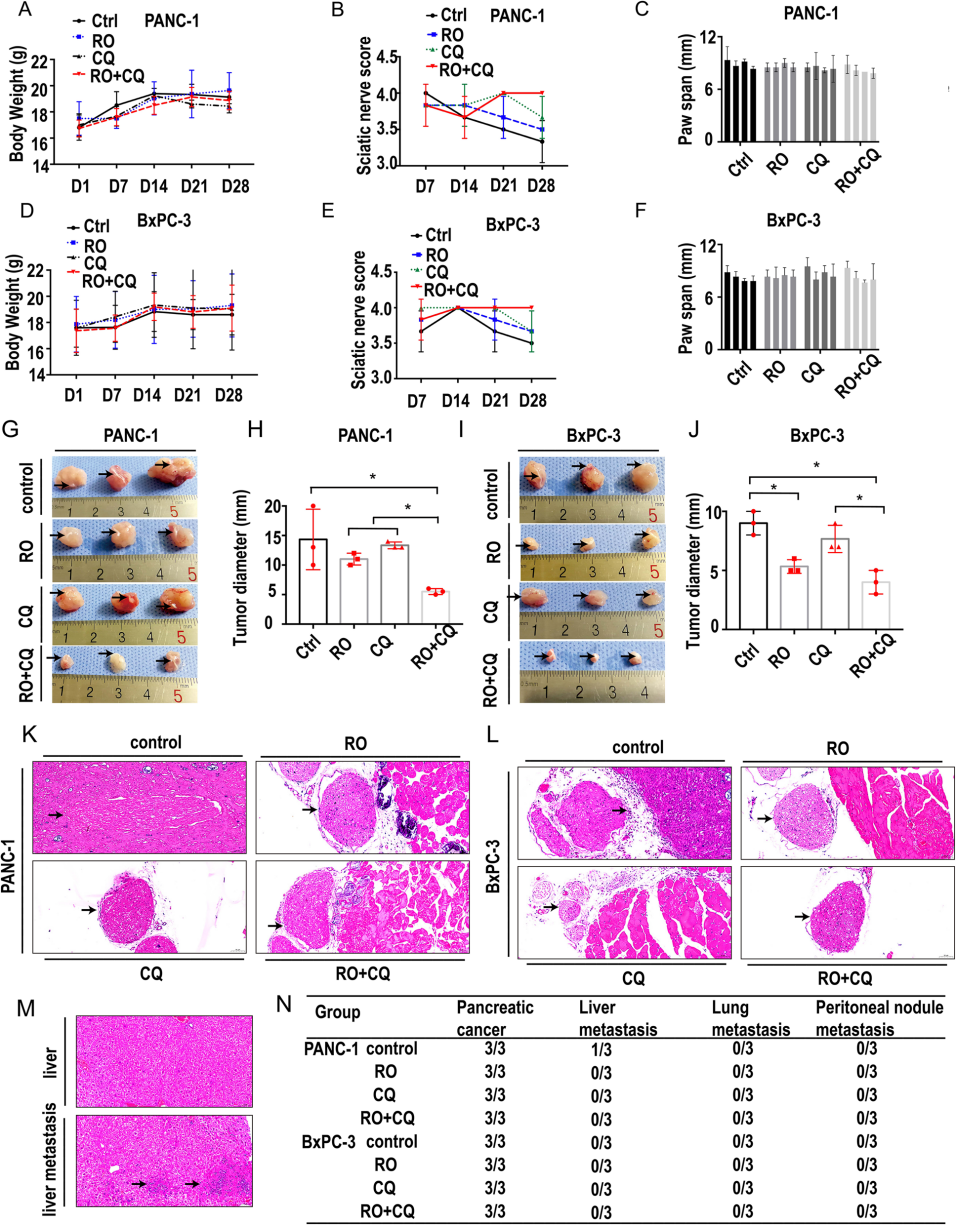
Double targeting of NGF and autophagy inhibits PNI in vivo. **A**. Body weight (g) of the nude mice with sciatic nerve PANC-1 injection treated with the control, RO, CQ or RO + CQ. No significant body weight difference was detected at day 1,7,14,21 and 28. **B**. Sciatic nerve score of the nude mice with sciatic nerve PANC-1 injection treated with the control, RO, CQ or RO + CQ. RO + CQ could increase the sciatic nerve score at day 28. **C**. The paw span (mm) of the nude mice with sciatic nerve PANC-1 injection treated with the control, RO, CQ or RO + CQ at day 28. **D**. Body weight (g) of the nude mice with sciatic nerve BxPC-3 injection treated with the control, RO, CQ or RO + CQ. No significant body weight difference was detected at day 1,7,14,21 and 28. **E**. Sciatic nerve score of the nude mice with sciatic nerve BxPC-3 injection treated with the control, RO, CQ or RO + CQ. RO + CQ could increase the sciatic nerve score at day 28. **F**. The paw span (mm) of the nude mice with sciatic nerve BxPC-3 injection treated with the control, RO, CQ or RO + CQ at day 28. **G**. PANC-1 nerve invasion specimens were collected on Day 28. Black arrow: sciatic nerve embedded in the tumor bulk. **H**. PANC-1 nerve invasion tumor size (mm) (tumor image: Fig. 7G) (* *p* < 0.05). **I**. BxPC-3 nerve invasion specimens were collected on Day 28. Black arrow: sciatic nerve embedded in the tumor bulk. **J**. BxPCc-3 nerve invasion tumor size (mm) (tumor image: Fig. 7I) (* *p* < 0.05). **K**. HE staining of the PANC-1 nerve invasion model. Black arrow: sciatic nerve. **L**. HE staining of the BxPC-3 nerve invasion model. Black arrow: sciatic nerve. **M**. HE staining of liver metastasis in the PANC-1 sciatic nerve invasion model. Black arrow: tumor metastasis. **N**. Tumor formation, liver metastasis, lung metastasis and peritoneal nodule metastasis status of the PANC-1 and BxPC-3 nerve invasion models

To confirm that the combination of an NGF inhibitor and chloroquine exerts antipancreatic cancer activity and PNI in vivo, we next examined its efficacy in PANC-1 and BxPC-3 sciatic nerve PNI models. Nude mice bearing PANC-1 and BxPC-3 cells were used as the sciatic nerve PNI model and were treated with vehicle (13.75 μg/g/3 days, 8 times,), RO 08–2750 (RO, 13.75 μg/g/3 days, 8 times, according to the manufacturer’s instruction), chloroquine (CQ, 10 μg/g/3 days, 8 times, according to previous study [19]) and RO + CQ (RO + CQ, 13.75 + 10 μg/g/3 days, 8 times) by intraperitoneal injection. As shown in Fig. 7A, the average body weights in the PANC-1 model were similar, indicating that host toxicity at these effective doses was acceptable. We examined sciatic nerve function, including the sciatic nerve score and the paw span distance (in mm) between the first and fifth toes, weekly. As shown in Fig. 7B, the hind limb paw response to manual extension was slower than that in the other groups without a significant difference. The paw span distance (in mm) between the first and fifth toes was also similar among all the groups on different days (Fig. 7C). We also tested the average body weights, sciatic nerve score and paw span distance (in mm) in the BxPC-3 model, and the findings were similar to the results in the PANC-1 model (Fig. 7D, E and F). The above results indicated that host toxicity at these effective doses was acceptable and that the combination of the NGF inhibitor and chloroquine in this PNI model had no effect on sciatic nerve functions.

All mice were euthanized at Day 28 after injection of cancer cells, and the sciatic nerve was excised for histopathologic analysis. Tumor diameter was measured at the injection site. For the PANC-1 model, the average long diameter of tumors in the RO + CQ group was 0.55 cm compared with that of the control (1.43 cm), RO (1.1 cm) and CQ (1.33 cm) groups (Fig. 7G). The sciatic nerve is indicated by the black arrow. The combination of the NGF inhibitor and chloroquine significantly decreased the tumor size compared with that of the other groups. The effect of the combination was significantly better than that of the NGF inhibitor or chloroquine alone (*p* < 0.05, Fig. 7H). For the BxPC-3 model, the average long diameter of tumors in the RO + CQ group was 0.40 cm compared with that of the control (0.90 cm) and CQ (0.77 cm) groups (Fig. 7I). The combination of the NGF inhibitor and chloroquine significantly decreased the tumor size compared with that of the control and CQ groups. The effect of the combination was significantly better than that of chloroquine alone (*p* < 0.05, Fig. 7J) and similar to that of the NGF inhibitor.

Histopathologic analysis showed that the tumor mass surrounded and entered the sciatic nerve in the PANC-1 model (Fig. 7K, arrow). There was some distance between the tumor and sciatic nerve in the other groups (Fig. 7K, arrow). For the BxPC-3 model, the outline of the sciatic nerve was changed, and the distance between the tumor and nerve was small (Fig. 7L, arrow), although the outline of the nerve was intact and there was some distance between the tumor and sciatic nerve in the other groups (Fig. 7L, arrow). Histopathologic analysis also showed the presence of liver metastasis in the control group of the PANC-1 model (Fig. 7M) compared with the other groups. Compared with the vehicle-treated mice, the treated mice showed inhibited metastasis to the liver in 1/3 (PANC-1). There was no metastasis to the liver in any of the groups in the BxPC-3 model. There was no metastasis to the lung or peritoneum in any of the groups (Fig. 7N).

### Schwann cell autophagy is inhibited by RO + CQ in vivo

**Fig. 8:**
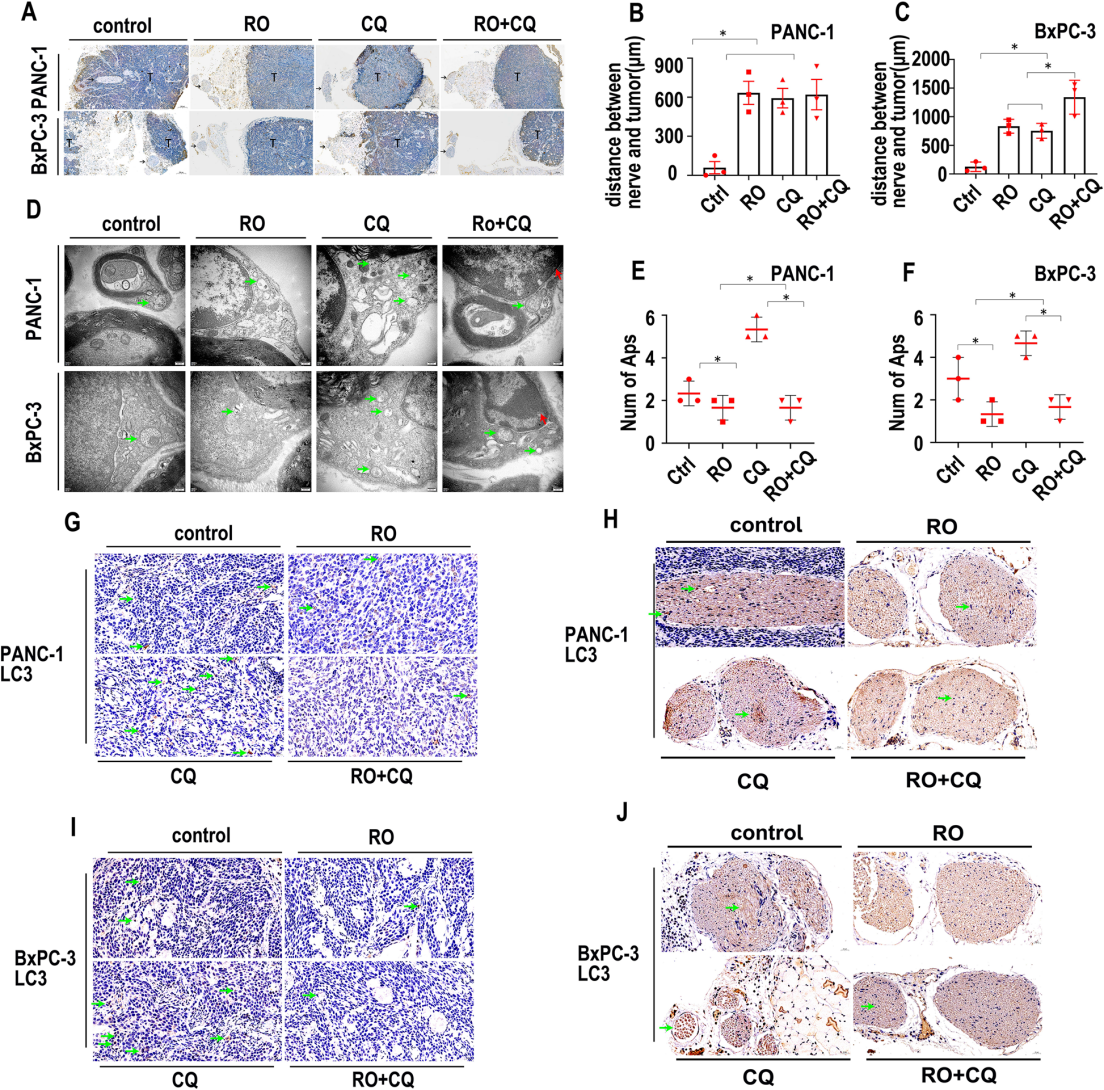
Schwann cell autophagy is inhibited by RO + CQ in vivo. **A**. CK19 IHC staining of the PANC-1 and BxPC-3 nerve invasion models. T indicates the tumor. Arrow indicates nerve. **B**. Statistics of the distance (μm) between nerves and tumors in the PANC-1 nerve invasion model (tumor image: Fig. 8A) (* *p* < 0.05). **C**. Statistics of the distance (μm) between nerves and tumors in the BxPC-3 nerve invasion model (tumor image: Fig. 8A) (* *p* < 0.05). **D**. TEM image of the PANC-1 and BxPC-3 nerve invasion models treated with the control, RO, CQ or RO + CQ. The green arrow indicates autophagosomes. The red arrow indicates apoptosis sign. **E**. Statistics of the autophagosome number of Schwann cells in the PANC-1 nerve invasion model treated with the control, RO, CQ or RO + CQ (cell image: Fig. 8D) (* *p* < 0.05). **F**. Statistics of the autophagosome number of Schwann cells in the BxPC-3 nerve invasion model treated with the control, RO, CQ or RO + CQ (cell image: Fig. 8D) (* *p* < 0.05). **G**. LC3 IHC staining of tumors in the PANC-1 nerve invasion model treated with the control, RO, CQ or RO + CQ. Green arrow indicates LC3-positive cancer cells. **H**. LC3 IHC staining of nerves in the PANC-1 nerve invasion model treated with the control, RO, CQ or RO + CQ. Green arrow indicates LC3-positive Schwann cells. **I**. LC3 IHC staining of tumors in the BxPC-3 nerve invasion model treated with the control, RO, CQ or RO + CQ. Green arrow indicates LC3-positive cancer cells. **J**. LC3 IHC staining of nerves in the BxPC-3 nerve invasion model treated with the control, RO, CQ or RO + CQ. Green arrow indicates LC3-positive Schwann cells

CK19 staining was used to mark cancer cells in IHC experiments. As shown in Fig. 8A, CK19 was positively expressed in cancer cells in the PANC-1 model, while CK19 was negatively expressed in nerves. The distances between nerves and tumors were significantly shorter in the control group than in the other groups (Fig. 8B), although the distances between nerves and tumors were similar in the three treatment groups without significant differences. For the BxPC-3 model, one of the tumor masses was near the nerves (Fig. 8A, arrow). The distances between nerves and tumors were significantly shorter in the control group than in the other groups (Fig. 8C). The distances between nerves and tumors were significantly shorter in the RO and CQ groups than in the RO + CQ group (Fig. 8C).

We next analyzed the morphological structures of the sciatic nerve by TEM. As shown in Fig. 8D, APs were observed in all the groups of the PANC-1 model (green arrowheads). Condensed chromatin edged around the nuclear membrane was found in the RO + CQ group (red arrowheads), although typical apoptotic bodies were not found in any of the groups. The number of APs in the CQ group was significantly greater than that in the other groups (Fig. 8E, *p* < 0.05).

For the BxPC-3 model, APs were also observed in all the groups (green arrowheads). Condensed chromatin edged around the nuclear membrane was found in the RO + CQ group (red arrowheads), although typical apoptotic bodies were not found in any of the groups. The number of APs in the CQ group was significantly greater than that in the other groups. The number of APs in the control group was significantly greater than that in the RO and RO + CQ groups (Fig. 8F, *p* < 0.05).

We next tested the expression of LC3 in the tumor mass and nerves. For the PANC-1 model, LC3 expression was positive around the nuclear membrane, and LC3 expression in the tumor masses was higher in the CQ group than in the other groups (Fig. 8G, green arrowheads). Positive expression of LC3 was also present in nerves, and higher aggregated expression was present in the CQ group than in the other groups (Fig. 8H, green arrowheads). Similar expression of LC3 was also present in the BxPC-3 model (Fig. 8I, J, green arrowheads). To explore whether the treatment can induce apoptosis, we performed IHC staining of caspase-3. RO and RO + CQ treatment induced the expression of caspase-3 in the tumor masses of the PANC-1 model (Fig. S7A, arrowhead), whereas RO + CQ treatment induced the expression of caspase-3 in the tumor masses of the BxPC-3 model (Fig. S7B, arrowhead). Apoptosis was not induced in the nerves in either of the models (Fig. S7C, D).

The above results showed that the combination of the NGF inhibitor and chloroquine can inhibit the growth of SCs and induce the apoptosis of cancer and then prevent the occurrence and development of perineural invasion in pancreatic cancer, which may be a potential treatment for PNI in PanCa.

**Fig. 9:**
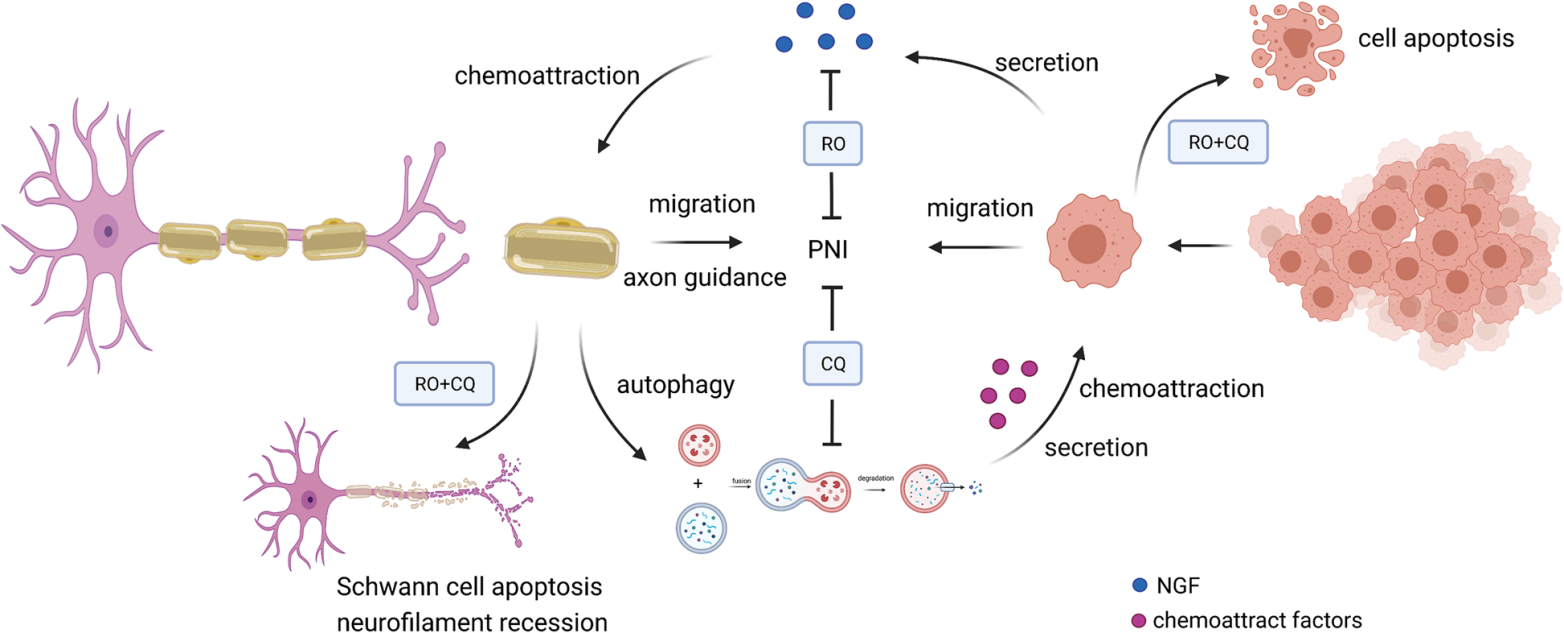
Schematic diagram for targeting NGF and autophagy in PNI treatment

The potential molecular mechanism by which autophagy of SCs promotes PNI in PanCa is that neurotrophic factors, such as NGF, secreted by cancer cells can induce autophagy of SCs by upregulating ATG7 expression. The autophagy of SCs can promote the outgrowth of nerve axons, which can act as a bridge in the occurrence and development of perineural invasion in pancreatic cancer (Fig. 9).

## Discussion

PanCa is characterized by infiltrating blood vessels, lymphatics and nerves. Perineural invasion (PNI), which has been reported in 70 to 100% of PanCa cases, is associated with peritoneal dissemination, tumor recurrence and worse Overall survival (OS), Disease-free survival (DFS)and progression-free survival (PFS) of PanCa [21–23]. PNI is a complex phenomenon that involves multidirectional communication of cells, including fibroblasts, pancreatic stellate cells, immune cells and Schwann cells, and molecules and pathways, including neurotrophic factors, neurotrophin receptors, chemokines, axonal guidance molecules, cellular adhesion molecules, matrix metalloproteinases and neurotransmitters between nerves and the cancer microenvironment [24].

The role of the nervous system has been recognized as an important contributor to cancer progression and metastasis by governing the functional activities of many organs in recent years [25]. Cancer cells can induce the outgrowth of nerves in the tumor microenvironment by the secretion of neurotrophic factors, such as nerve growth factor, and in turn nerves are emerging regulators of cancer initiation, progression, and metastasis [26]. Pancreatic acinar-derived cells can invade along sensory neurons into the spinal cord and migrate to the lower thoracic and upper lumbar regions at the PanIN2 stage. This effect was prevented by sensory neuron ablation by neonatal capsaicin injection, indicating that sensory neurons may represent an important stromal cell in the initiation and progression of PanCa [27]. Increasing evidence indicates that nerves can interact with cancer cells through two-way communication through active inputs to tumors and dynamically exert significant control over cancer progression [28].

Autophagy can participate in some important cellular processes, such as metabolic reprogramming, cell death, immune evasion, and metastasis, which contribute to tumor development [29, 30], and is also related to resistance to chemotherapies and targeted therapies in cancer via various signaling pathways, including PI3K/AKT and MAPK signaling [31]. A high level of autophagy is found in the basal state in PanCa cell lines and tumor tissues and is important in the progression of PanCa by modulating invasion and metastasis, proliferation, cell death, metabolism, or immunity [32]. Autophagy is also related to resistance to cytotoxic chemotherapy and targeted therapy in PanCa and other cancers [33].

The relationship between autophagy and pancreatic stroma is also important in the progression of PanCa. The higher autophagic level of pancreatic stellate cells (PSCs) in PanCa tissue leads to higher expression of extracellular matrix and proinflammatory IL-6 (interleukin 6) in PSCs [34]. High LC3 expression is related to PNI and is an independent risk factor for the poor prognosis of PanCa [35], but the concrete mechanism is not clear. We also found that LC3 expression was even stronger in nerves than in PanCa tissue and had the same position as GFAP staining-positive SCs, indicating that the interaction between cancer cells and nerves can promote autophagy in SCs. Our in vitro results showed that conditioned medium (CM1 and CM2) from PanCa cells increased the expression of the autophagy-initiating proteins p-mTOR, p-ULK1 and ATG5. The ratio of LC3II to LC3I increased in the CM groups, and P62 expression decreased in the CM1 and CM2 groups, indicating that pancreatic cancer can activate autophagy by a paracrine mechanism related to PNI in PanCa. Here, we report that pancreatic cancer cells can activate Schwann cell autophagy in a paracrine manner in vitro.

Schwann cells can also be detected around human and murine pancreatic intraepithelial neoplasias (PanINs) and intestinal adenomas and also migrate toward PanCa cells before the cancer cells migrate toward peripheral nerves but not toward benign cells, indicating specific affinity of Schwann cells and cancer cells [36]. The tumor-neuroglia interaction is activated by coculture of SCs with PanCa cells, in which interleukin 1β (IL1β) is secreted by tumor cells and then activates the nuclear actor (NF)-kappa B pathway in SCs, resulting in increased interleukin 6 (IL-6) and promoting cancer cell migration and invasion by activating STAT3 signaling in cancer cells [37]. SC-derived proteins, including matrix metalloproteinase-2, cathepsin D, plasminogen activator inhibitor-1, and galectin-1, are related to the proliferation and invasion of PanCa cells [12].

SCs can promote neurite outgrowth by direct contact with neurites in addition to secreting factors during coculture with adult SCs for peripheral nervous system injury [38]. CXCL12 can induce Schwann cell autophagy and migration by the PI3K-AKT-mTOR signaling pathway after facial nerve injury, which is useful for myelin regeneration [39]. Myelin degradation can activate autophagy in SCs, which is a ubiquitous cytoprotective process and essential for degrading and recycling cellular constituents [15], indicating that autophagy in SCs is important in the repair and regeneration of nerve injury. Autophagy of SCs for PNI in PanCa is unclear. In our in vitro experiment, after ATG7 in SCs was knocked down by shRNA, the proliferation of SCs was inhibited significantly; however, the migration of SCs was not changed in a two-dimensional environment but was affected in a 3D environment. PANC-1 and BxPC-3 CM also promoted SC migration in DRG cultures.

Increased expression of NGF/TrkA may contribute to perineural invasion and pain syndrome in PanCa [40]. Artemin, NGF and growth-associated protein-43 expression was increased in both the histologically “normal” pancreatic parenchyma next to PanCa and PanCa tissue with dense neural networks and enlarged nerves, revealing the effect of NGF as a key player in the generation of pancreatic neuropathy in PanCa [41]. NGF may contribute to PNI by restraining the apoptosis of tumor cells, promoting the hyperplasia of nerves, and specifically enhancing the NGF and TrkA interaction [42]. Enlarged nerves and dense neural networks are detected in PanCa tissue and histologically “normal” pancreatic parenchyma next to PanCa, which is related to intrapancreatic neuropathy. Nerve growth factor (NGF) may be a potential key player in the generation of pancreatic neuropathy in PanCa [41]. NGF/tropomyosin-related kinase A (TrkA) promotes PanCa cell proliferation and invasion in a pancreatic stellate cell-pancreatic cancer cell coculture system via activation of PI3K/AKT/GSK signaling, which may be a potential therapeutic target for PC patients [43].

Growth factors derived from SCs, such as NGF, can promote the neurite outgrowth of DRG neurons [44]. The autophagy of SCs can be activated by exogenous NGF, which is helpful for phagocytosis and myelin debris clearance. Axon and myelin regeneration are also promoted at the early stage of peripheral nerve injury, and the p75NTR/AMPK/mTOR axis is probably involved in this regulation [16]. We found that NGF secreted by PanCa cells can mimic the effect of exogenous NGF, induce the autophagy of SCs, and then promote PNI via the proliferation of SCs and cancer cells and the outgrowth of nerve fibers. Knocking down NGF partially abolished the autophagic activating effect, in which the p-mTOR level was inhibited while the p-ULK1 level of SCs was elevated after coculture with PanCa cell line-conditioned medium. Knockdown of NGF in the PanCa cell line partially reversed this phenomenon. The p-AMPK level was not detected in our research. The exact molecular mechanism still needs further exploration for confirmation.

As inhibitors of autophagy, chloroquine and hydroxychloroquine can modulate autophagy and have been tested in clinical studies of PanCa patients. Single-agent hydroxychloroquine in metastatic PanCa did not show a response [45]. CQ impairs the fusion of autophagosomes (LAMP2-negative SQSTM1 puncta) with lysosomes and the content of LAMP2-labeled DGCs was condensed [19]. The authors also indicate that CQ treatment could increase the area of LAMP1-positive structures(late endosomes and lysosomes) and the average span of the lysosomes. The authors describe DGC (degradative compartments) including lysosomes, autolysosomes and amphisomes under CQ treatment in TEM as ‘condensed amorphous contents’,which is similar to our observation in CQ treated RSC96 cells. We describe these high density structure as “dysfunctional lysosomes” to emphasize that CQ treatment could inhibit the proper function of lysosomes in the autophagy process. The combination of chloroquine or hydroxychloroquine with chemotherapy has also shown inconsistent results [33].

The gold nanocluster-assisted delivery of NGF siRNA (GNC-siRNA) is used to inhibit tumor progression in subcutaneous models, orthotopic models and patient-derived xenograft models [46]. It has been reported that ablation of specific nerve types (parasympathetic, sympathetic, or sensory) inhibits tumor growth in a tissue-specific manner [47]. Overexpression of NGF correlates with a poorer prognosis, perineural invasion and pain severity. Anti-NGF treatment at different times can influence neural inflammation, neural invasion, and metastasis [48]. Knocking down NGF or its receptors can reduce the proliferation and migration of PanCa cells, and the migratory ability of Mia PaCa2 cells toward the DRG indicates a potential target for developing molecularly targeted therapies to decrease PNI [49]. Our results showed that targeting NGF and autophagy inhibited proliferation and invasion by inhibiting autophagic initiation and autophagic flux and caused apoptosis of SCs, cancer cells and DRGs in vitro. The combination of an NGF inhibitor and chloroquine can prevent the occurrence and development of perineural invasion in pancreatic cancer, and potential molecular neurotrophic factors, such as NGF, secreted by cancer cells can induce autophagy of SCs by upregulating ATG7 expression.

Thus, autophagy in SCs can be induced by through Panca paracrine pathways such as the NGF/ATG7 pathway. Autophagic SCs exert a “nerve-repair like effect” on nerves and promote the extension of nerve fibers towards PanCa cells. Moreover, autophagic SCs promotes the aggressiveness of cancer cells especially the extensional chemotaxis towards SCs and nerve, providing a “beacon” for the invasion of cancer cells to nerve fibers and directional growth of cancer cells.

## Conclusions

PanCa cells can induce autophagy in SCs through paracrine pathways such as the NGF/ATG7 pathway. Autophagic SCs exert a “nerve-repair like effect”, induce a high level of autophagy of cancer cells, provide a “beacon” for the invasion of cancer cells to nerve fibers, and induce directional growth of cancer cells. Targeting NGF and autophagy for PNI treatment can block nerve infiltration and is expected to provide new directions and an experimental basis for the research and treatment of nerve infiltration in pancreatic cancer.

## Supplementary Information

**Additional file 1: Fig. S1.** A. Identification of Schwann cells in DRG culture. Blue: DAPI labeling of nucleus. Green: GFAP. Red: NF-H. Schwann cells are both GFAP-positive and NF-H-positive. B. Identification of Schwann cells and neurofilaments in DRG culture. Red arrow: neurofilament labeled with NF09 or NF-H. Yellow arrow: Schwann cells. C. PanCa-conditioned medium (CM) activates Schwann cell autophagy in the DRG-PanCa coculture system. Blue: DAPI-labeled cell nuclei. Red: LC3. Green arrow: autophagy-activated Schwann cells with LC3 puncta. Yellow arrow: autophagy unactivated Schwann cells. D. Statistics of autophagic and non-autophagic Schwann cells in the DRG cultured with PanCa CM (cell image: Fig. S1C) (* *p* < 0.05). E. ATG5 immunofluorescence staining of Schwann cells treated with PanCa CM. F. Statistics of ATG5 positive and negative RSC96 cells treated with PanCa CM (cell image: Fig. S1E) (* *p* < 0.05).

**Additional file 2: Fig. S2.** A. Western blotting of NGF in the control or NGF knockdown PDAC cell lines. GAPDH was used as a loading control. B. NGF protein expression in PanCa and shNGF-PanCa cell lines in PANC-1 and BxPC-3 cell lines. C. NGF (50 ng/ml) induces the P62 and LC3 puncta accumulation in RSC96 cell line. D. Statistic of autophasosomes and autophagolysosomes in RSC96 treated with NGF (50 ng/ml) or control. (cell image: Fig. S2E). E. Autophagic flux detection of RSC96 cells treated with the control or NGF (50 ng/ml). NGF promotes autophagy flux in RSC96 cells.

**Additional file 3: Fig. S3.** A. Western blotting of ATG7 protein expression of RSC96 and shATG7-RSC96 cells. B. ATG7 protein expression of RSC96 and shATG7-RSC96 cells. C. Statistics of the RSC96 and shATG7-RSC96 migration treated with the control, NGF, PANC-1 CM or BxPC-3 CM. (cell image: Fig. S3D). D. Wound healing assays for RSC96 and shATG7-RSC96 cells treated with the control, NGF, PANC-1 CM or BxPC-3 CM. E. Transwell migration assays of RSC96 and shATG7-RSC96 cells treated with the control or NGF, PanCa CM or shNGF-PanCa CM.

**Additional file 4: Fig. S4.** A. Light microscopy of RSC96, PANC-1 and BxPC-3 cells treated with the control, RO, CQ or RO + CQ for 24 h. The green arrow indicates normal cells, and the red arrow indicates apoptotic cells. B. Statistics of apoptotic cells treated with ctrl, RO, CQ or RO+CQ (cell image: Fig. S4A). C. Cleaved PARP1 immunofluorescence (red) of RSC96 cells treated with RO, CQ or RO + CQ. Yellow arrow: cleaved PARP1- positive cell. Green arrow: cleaved PARP1-negative cell. D. Cleaved caspase-3 immunofluorescence (red) of RSC96 cells treated with RO, CQ or RO + CQ. Yellow arrow: cleaved caspase-3-positive cells. Green arrow: cleaved caspase-3-negative cells.

**Additional file 5: Fig. S5.** A. LC3 immunofluorescence of PANC-1 cells treated with RO, CQ or RO + CQ. White arrow: LC3 positive puncta. B. P62 immunofluorescence of PANC-1 cells treated with RO, CQ or RO + CQ. White arrow: P62 positive puncta. C. LC3 immunofluorescence of BxPC-3 cells treated with RO, CQ or RO + CQ. White arrow: LC3 positive puncta. D. P62 immunofluorescence of BxPC-3 cells treated with RO, CQ or RO + CQ. White arrow: P62 positive puncta. E. Cleaved PARP1 immunofluorescence (red) of PANC-1 cells treated with RO, CQ or RO + CQ. Yellow arrow: cleaved PARP1-negative cells. Green arrow: cleaved PARP1-positive cells. F. Cleaved caspase-3 immunofluorescence (red) of PANC-1 cells treated with RO, CQ or RO + CQ. Yellow arrow: cleaved caspase-3-negative cells. Green arrow: cleaved caspase-3-positive cells. G. Cleaved PARP1 immunofluorescence (red) of BxPC-3 cells treated with RO, CQ or RO + CQ. Yellow arrow: cleaved PARP1-negative cells. Green arrow: cleaved PARP1-positive cells. H. Cleaved caspase-3 immunofluorescence (red) of BxPC-3 cells treated with RO, CQ or RO + CQ. Yellow arrow: cleaved caspase-3-negative cells. Green arrow: cleaved caspase-3-positive cells.

**Additional file 6: Fig. S6**. A. LC3 (red) and NF09 (green) double immunofluorescence of DRG monoculture treated with the control, RO, CQ or RO + CQ. B. Cleaved caspase-3 (red) immunofluorescence of DRG monoculture treated with the control, RO, CQ or RO + CQ. The green arrow indicates cleaved caspase-3-positive Schwann cells. C. Cleaved caspase-3 (red) and NF09 (green) double immunofluorescence of the DRG and BxPC-3 coculture system treated with the control, RO, CQ or RO + CQ. D. Cleaved caspase-3 (red) and NF09 (green) double immunofluorescence of the DRG and PANC-1 coculture system treated with the control, RO, CQ or RO + CQ. E. LC3 (red) and NF09 (green) double immunofluorescence of DRG and BxPC-3 coculture systems treated with the control, RO, CQ or RO + CQ. F. LC3 (red) and NF09 (green) double immunofluorescence of the DRG and PANC-1 coculture system treated with the control, RO, CQ or RO + CQ.

**Additional file 7: Fig. S7.** A. Cleaved caspase-3 IHC staining of tumors in the PANC-1 nerve invasion model treated with the control, RO, CQ or RO + CQ. Black arrow indicates cleaved caspase-3-positive cancer cells. B. Cleaved caspase-3 IHC staining of tumors in the BxPC-3 nerve invasion model treated with the control, RO, CQ or RO + CQ. Black arrow indicates cleaved caspase-3-positive cancer cells. C. Cleaved caspase-3 IHC staining of nerves in the PANC-1 nerve invasion model treated with the control, RO, CQ or RO + CQ. D. Cleaved caspase-3 IHC staining of nerves in the BxPC-3 nerve invasion model treated with the control, RO, CQ or RO + CQ.

**Additional file 8: Fig. S8.** A. Statistic graph of Western blotting of Fig. 1M. B. Statistic graph of Western blotting of Fig. 2N. C. Statistic graph of Western blotting of Fig. 2O. D. Statistic graph of Western blotting of Fig. 4K. E. Statistic graph of Western blotting of Fig. 5N.

## Data Availability

Not applicable.

## Abbreviations

PNI: Perineural invasion
PanCa: Pancreatic cancer
SCs: Schwann cells
DRG: Dorsal root ganglion
TEM: Transmission electron microscopy
RO: RO 08–2750
CQ: Chloroquine
EU: European Union
PanCa: Pancreatic cancer
NGF: Nerve growth factor
CM: Conditioned medium

## References

[1] Maisonneuve P (2019). Epidemiology and burden of pancreatic cancer. Presse Med.

[2] Siegel RL, Miller KD, Fuchs HE, Jemal A (2021). Cancer Statistics, 2021. CA Cancer J Clin.

[3] Khalaf N, El-Serag HB, Abrams HR, Thrift AP (2021). Burden of Pancreatic Cancer: From Epidemiology to Practice. Clin Gastroenterol Hepatol.

[4] Rahib L, Smith BD, Aizenberg R, Rosenzweig AB, Fleshman JM, Matrisian LM (2014). Projecting cancer incidence and deaths to 2030: the unexpected burden of thyroid, liver, and pancreas cancers in the United States. Cancer Res.

[5] Alrawashdeh W, Jones R, Dumartin L, Radon TP, Cutillas PR, Feakins RM, Dmitrovic B, Demir IE, Ceyhan GO, Crnogorac-Jurcevic T (2019). Perineural invasion in pancreatic cancer: proteomic analysis and in vitro modelling. Mol Oncol.

[6] Chatterjee D, Katz MH, Rashid A, Wang H, Iuga AC, Varadhachary GR, Wolff RA, Lee JE, Pisters PW, Crane CH, Gomez HF, Abbruzzese JL, Fleming JB, Wang H (2012). Perineural and intraneural invasion in posttherapy pancreaticoduodenectomy specimens predicts poor prognosis in patients with pancreatic ductal adenocarcinoma. Am J Surg Pathol.

[7] Zahalka AH, Frenette PS (2020). Nerves in cancer. Nat Rev Cancer.

[8] Liao C-P, Booker RC, Brosseau J-P, Chen Z, Mo J, Tchegnon E, Wang Y, Clapp DW, Le LQ (2018). Contributions of inflammation and tumor microenvironment to neurofibroma tumorigenesis. J Clin Invest.

[9] Deborde S, Omelchenko T, Lyubchik A, Zhou Y, He S, McNamara WF, Chernichenko N, Lee S-Y, Barajas F, Chen C-H, Bakst RL, Vakiani E, He S, Hall A, Wong RJ (2016). Schwann cells induce cancer cell dispersion and invasion. J Clin Invest.

[10] Deborde S, Wong RJ (2017). How Schwann cells facilitate cancer progression in nerves. Cell Mol Life Sci.

[11] Fujii-Nishimura Y, Yamazaki K, Masugi Y, Douguchi J, Kurebayashi Y, Kubota N, Ojima H, Kitago M, Shinoda M, Hashiguchi A, Sakamoto M (2018). Mesenchymal-epithelial transition of pancreatic cancer cells at perineural invasion sites is induced by Schwann cells. Pathol Int.

[12] Ferdoushi A, Li X, Griffin N, Faulkner S, Jamaluddin MFB, Gao F, Jiang CC, van Helden DF, Tanwar PS, Jobling P, Hondermarck H (2020). Schwann Cell Stimulation of Pancreatic Cancer Cells: A Proteomic Analysis. Front Oncol.

[13] Namgung U (2014). The role of Schwann cell-axon interaction in peripheral nerve regeneration. Cells Tissues Organs.

[14] Hashimoto D, Blauer M, Hirota M, Ikonen NH, Sand J, Laukkarinen J (2014). Autophagy is needed for the growth of pancreatic adenocarcinoma and has a cytoprotective effect against anticancer drugs. Eur J Cancer.

[15] Ko PY, Yang CC, Kuo YL, Su FC, Hsu TI, Tu YK, Jou IM (2018). Schwann-Cell Autophagy, Functional Recovery, and Scar Reduction After Peripheral Nerve Repair. J Mol Neurosci.

[16] Li R, Li D, Wu C, Ye L, Wu Y, Yuan Y, Yang S, Xie L, Mao Y, Jiang T, Li Y, Wang J, Zhang H, Li X, Xiao J (2020). Nerve growth factor activates autophagy in Schwann cells to enhance myelin debris clearance and to expedite nerve regeneration. Theranostics.

[17] Li J, Ma J, Han L, Xu Q, Lei J, Duan W, Li W, Wang F, Wu E, Ma Q, Huo X (2015). Hyperglycemic tumor microenvironment induces perineural invasion in pancreatic cancer. Cancer Biol Ther.

[18] Wang B, Zhong Y, Li Q, Cui L, Huang G (2018). Autophagy of macrophages is regulated by PI3k/Akt/mTOR signalling in the development of diabetic encephalopathy. Aging (Albany NY).

[19] Mauthe M, Orhon I, Rocchi C, Zhou X, Luhr M, Hijlkema K-J, Coppes RP, Engedal N, Mari M, Reggiori F (2018). Chloroquine inhibits autophagic flux by decreasing autophagosome-lysosome fusion. Autophagy.

[20] Klionsky DJ, Abdalla FC, Abeliovich H, Abraham RT, Acevedo-Arozena A, Adeli K, Agholme L, Agnello M, Agostinis P, Aguirre-Ghiso JA, Ahn HJ, Ait-Mohamed O, Ait-Si-Ali S, Akematsu T, Akira S, Al-Younes HM, Al-Zeer MA, Albert ML, Albin RL, Alegre-Abarrategui J, Aleo MF, Alirezaei M, Almasan A, Almonte-Becerril M, Amano A, Amaravadi R, Amarnath S, Amer AO, Andrieu-Abadie N, Anantharam V, Ann DK, Anoopkumar-Dukie S, Aoki H, Apostolova N, Arancia G, Aris JP, Asanuma K, Asare NYO, Ashida H, Askanas V, Askew DS, Auberger P, Baba M, Backues SK, Baehrecke EH, Bahr BA, Bai X-Y, Bailly Y, Baiocchi R, Baldini G, Balduini W, Ballabio A, Bamber BA, Bampton ETW, Bánhegyi G, Bartholomew CR, Bassham DC, Bast RC, Batoko H, Bay B-H, Beau I, Béchet DM, Begley TJ, Behl C, Behrends C, Bekri S, Bellaire B, Bendall LJ, Benetti L, Berliocchi L, Bernardi H, Bernassola F, Besteiro S, Bhatia-Kissova I, Bi X, Biard-Piechaczyk M, Blum JS, Boise LH, Bonaldo P, Boone DL, Bornhauser BC, Bortoluci KR, Bossis I, Bost F, Bourquin J-P, Boya P, Boyer-Guittaut M, Bozhkov PV, Brady NR, Brancolini C, Brech A, Brenman JE, Brennand A, Bresnick EH, Brest P, Bridges D, Bristol ML, Brookes PS, Brown EJ, Brumell JH, Brunetti-Pierri N, Brunk UT, Bulman DE, Bultman SJ, Bultynck G, Burbulla LF, Bursch W, Butchar JP, Buzgariu W, Bydlowski SP, Cadwell K, Cahová M, Cai D, Cai J, Cai Q, Calabretta B, Calvo-Garrido J, Camougrand N, Campanella M, Campos-Salinas J, Candi E, Cao L, Caplan AB, Carding SR, Cardoso SM, Carew JS, Carlin CR, Carmignac V, Carneiro LAM, Carra S, Caruso RA, Casari G, Casas C, Castino R, Cebollero E, Cecconi F, Celli J, Chaachouay H, Chae H-J, Chai C-Y, Chan DC, Chan EY, Chang RC-C, Che C-M, Chen C-C, Chen G-C, Chen G-Q, Chen M, Chen Q, Chen SSL, Chen W, Chen X, Chen X, Chen X, Chen Y-G, Chen Y, Chen Y, Chen Y-J, Chen Z, Cheng A, Cheng CHK, Cheng Y, Cheong H, Cheong J-H, Cherry S, Chess-Williams R, Cheung ZH, Chevet E, Chiang H-L, Chiarelli R, Chiba T, Chin L-S, Chiou S-H, Chisari FV, Cho CH, Cho D-H, Choi AMK, Choi D, Choi KS, Choi ME, Chouaib S, Choubey D, Choubey V, Chu CT, Chuang T-H, Chueh S-H, Chun T, Chwae Y-J, Chye M-L, Ciarcia R, Ciriolo MR, Clague MJ, Clark RSB, Clarke PGH, Clarke R, Codogno P, Coller HA, Colombo MI, Comincini S, Condello M, Condorelli F, Cookson MR, Coombs GH, Coppens I, Corbalan R, Cossart P, Costelli P, Costes S, Coto-Montes A, Couve E, Coxon FP, Cregg JM, Crespo JL, Cronjé MJ, Cuervo AM, Cullen JJ, Czaja MJ, D'Amelio M, Darfeuille-Michaud A, Davids LM, Davies FE, De Felici M, de Groot JF, de Haan CAM, De Martino L, De Milito A, De Tata V, Debnath J, Degterev A, Dehay B, Delbridge LMD, Demarchi F, Deng YZ, Dengjel J, Dent P, Denton D, Deretic V, Desai SD, Devenish RJ, Di Gioacchino M, Di Paolo G, Di Pietro C, Díaz-Araya G, Díaz-Laviada I, Diaz-Meco MT, Diaz-Nido J, Dikic I, Dinesh-Kumar SP, Ding W-X, Distelhorst CW, Diwan A, Djavaheri-Mergny M, Dokudovskaya S, Dong Z, Dorsey FC, Dosenko V, Dowling JJ, Doxsey S, Dreux M, Drew ME, Duan Q, Duchosal MA, Duff K, Dugail I, Durbeej M, Duszenko M, Edelstein CL, Edinger AL, Egea G, Eichinger L, Eissa NT, Ekmekcioglu S, El-Deiry WS, Elazar Z, Elgendy M, Ellerby LM, Eng KE, Engelbrecht A-M, Engelender S, Erenpreisa J, Escalante R, Esclatine A, Eskelinen E-L, Espert L, Espina V, Fan H, Fan J, Fan Q-W, Fan Z, Fang S, Fang Y, Fanto M, Fanzani A, Farkas T, Farré J-C, Faure M, Fechheimer M, Feng CG, Feng J, Feng Q, Feng Y, Fésüs L, Feuer R, Figueiredo-Pereira ME, Fimia GM, Fingar DC, Finkbeiner S, Finkel T, Finley KD, Fiorito F, Fisher EA, Fisher PB, Flajolet M, Florez-McClure ML, Florio S, Fon EA, Fornai F, Fortunato F, Fotedar R, Fowler DH, Fox HS, Franco R, Frankel LB, Fransen M, Fuentes JM, Fueyo J, Fujii J, Fujisaki K, Fujita E, Fukuda M, Furukawa RH, Gaestel M, Gailly P, Gajewska M, Galliot B, Galy V, Ganesh S, Ganetzky B, Ganley IG, Gao F-B, Gao GF, Gao J, Garcia L, Garcia-Manero G, Garcia-Marcos M, Garmyn M, Gartel AL, Gatti E, Gautel M, Gawriluk TR, Gegg ME, Geng J, Germain M, Gestwicki JE, Gewirtz DA, Ghavami S, Ghosh P, Giammarioli AM, Giatromanolaki AN, Gibson SB, Gilkerson RW, Ginger ML, Ginsberg HN, Golab J, Goligorsky MS, Golstein P, Gomez-Manzano C, Goncu E, Gongora C, Gonzalez CD, Gonzalez R, González-Estévez C, González-Polo RA, Gonzalez-Rey E, Gorbunov NV, Gorski S, Goruppi S, Gottlieb RA, Gozuacik D, Granato GE, Grant GD, Green KN, Gregorc A, Gros F, Grose C, Grunt TW, Gual P, Guan J-L, Guan K-L, Guichard SM, Gukovskaya AS, Gukovsky I, Gunst J, Gustafsson AB, Halayko AJ, Hale AN, Halonen SK, Hamasaki M, Han F, Han T, Hancock MK, Hansen M, Harada H, Harada M, Hardt SE, Harper JW, Harris AL, Harris J, Harris SD, Hashimoto M, Haspel JA, Hayashi S-i, Hazelhurst LA, He C, He Y-W, Hébert M-J, Heidenreich KA, Helfrich MH, Helgason GV, Henske EP, Herman B, Herman PK, Hetz C, Hilfiker S, Hill JA, Hocking LJ, Hofman P, Hofmann TG, Höhfeld J, Holyoake TL, Hong M-H, Hood DA, Hotamisligil GS, Houwerzijl EJ, Høyer-Hansen M, Hu B, Hu C-AA, Hu H-M, Hua Y, Huang C, Huang J, Huang S, Huang W-P, Huber TB, Huh W-K, Hung T-H, Hupp TR, Hur GM, Hurley JB, Hussain SNA, Hussey PJ, Hwang JJ, Hwang S, Ichihara A, Ilkhanizadeh S, Inoki K, Into T, Iovane V, Iovanna JL, Ip NY, Isaka Y, Ishida H, Isidoro C, Isobe K-i, Iwasaki A, Izquierdo M, Izumi Y, Jaakkola PM, Jäättelä M, Jackson GR, Jackson WT, Janji B, Jendrach M, Jeon J-H, Jeung E-B, Jiang H, Jiang H, Jiang JX, Jiang M, Jiang Q, Jiang X, Jiang X, Jiménez A, Jin M, Jin S, Joe CO, Johansen T, Johnson DE, Johnson GVW, Jones NL, Joseph B, Joseph SK, Joubert AM, Juhász G, Juillerat-Jeanneret L, Jung CH, Jung Y-K, Kaarniranta K, Kaasik A, Kabuta T, Kadowaki M, Kagedal K, Kamada Y, Kaminskyy VO, Kampinga HH, Kanamori H, Kang C, Kang KB, Kang KI, Kang R, Kang Y-A, Kanki T, Kanneganti T-D, Kanno H, Kanthasamy AG, Kanthasamy A, Karantza V, Kaushal GP, Kaushik S, Kawazoe Y, Ke P-Y, Kehrl JH, Kelekar A, Kerkhoff C, Kessel DH, Khalil H, Kiel JAKW, Kiger AA, Kihara A, Kim DR, Kim D-H, Kim D-H, Kim E-K, Kim H-R, Kim J-S, Kim JH, Kim JC, Kim JK, Kim PK, Kim SW, Kim Y-S, Kim Y, Kimchi A, Kimmelman AC, King JS, Kinsella TJ, Kirkin V, Kirshenbaum LA, Kitamoto K, Kitazato K, Klein L, Klimecki WT, Klucken J, Knecht E, Ko BCB, Koch JC, Koga H, Koh J-Y, Koh YH, Koike M, Komatsu M, Kominami E, Kong HJ, Kong W-J, Korolchuk VI, Kotake Y, Koukourakis MI, Kouri Flores JB, Kovács AL, Kraft C, Krainc D, Krämer H, Kretz-Remy C, Krichevsky AM, Kroemer G, Krüger R, Krut O, Ktistakis NT, Kuan C-Y, Kucharczyk R, Kumar A, Kumar R, Kumar S, Kundu M, Kung H-J, Kurz T, Kwon HJ, La Spada AR, Lafont F, Lamark T, Landry J, Lane JD, Lapaquette P, Laporte JF, László L, Lavandero S, Lavoie JN, Layfield R, Lazo PA, Le W, Le Cam L, Ledbetter DJ, Lee AJX, Lee B-W, Lee GM, Lee J, Lee J-H, Lee M, Lee M-S, Lee SH, Leeuwenburgh C, Legembre P, Legouis R, Lehmann M, Lei H-Y, Lei Q-Y, Leib DA, Leiro J, Lemasters JJ, Lemoine A, Lesniak MS, Lev D, Levenson VV, Levine B, Levy E, Li F, Li J-L, Li L, Li S, Li W, Li X-J, Li Y-b, Li Y-P, Liang C, Liang Q, Liao Y-F, Liberski PP, Lieberman A, Lim HJ, Lim K-L, Lim K, Lin C-F, Lin F-C, Lin J, Lin JD, Lin K, Lin W-W, Lin W-C, Lin Y-L, Linden R, Lingor P, Lippincott-Schwartz J, Lisanti MP, Liton PB, Liu B, Liu C-F, Liu K, Liu L, Liu QA, Liu W, Liu Y-C, Liu Y, Lockshin RA, Lok C-N, Lonial S, Loos B, Lopez-Berestein G, López-Otín C, Lossi L, Lotze MT, Lőw P, Lu B, Lu B, Lu B, Lu Z, Luciano F, Lukacs NW, Lund AH, Lynch-Day MA, Ma Y, Macian F, MacKeigan JP, Macleod KF, Madeo F, Maiuri L, Maiuri MC, Malagoli D, Malicdan MCV, Malorni W, Man N, Mandelkow E-M, Manon S, Manov I, Mao K, Mao X, Mao Z, Marambaud P, Marazziti D, Marcel YL, Marchbank K, Marchetti P, Marciniak SJ, Marcondes M, Mardi M, Marfe G, Mariño G, Markaki M, Marten MR, Martin SJ, Martinand-Mari C, Martinet W, Martinez-Vicente M, Masini M, Matarrese P, Matsuo S, Matteoni R, Mayer A, Mazure NM, McConkey DJ, McConnell MJ, McDermott C, McDonald C, McInerney GM, McKenna SL, McLaughlin B, McLean PJ, McMaster CR, McQuibban GA, Meijer AJ, Meisler MH, Meléndez A, Melia TJ, Melino G, Mena MA, Menendez JA, Menna-Barreto RFS, Menon MB, Menzies FM, Mercer CA, Merighi A, Merry DE, Meschini S, Meyer CG, Meyer TF, Miao C-Y, Miao J-Y, Michels PAM, Michiels C, Mijaljica D, Milojkovic A, Minucci S, Miracco C, Miranti CK, Mitroulis I, Miyazawa K, Mizushima N, Mograbi B, Mohseni S, Molero X, Mollereau B, Mollinedo F, Momoi T, Monastyrska I, Monick MM, Monteiro MJ, Moore MN, Mora R, Moreau K, Moreira PI, Moriyasu Y, Moscat J, Mostowy S, Mottram JC, Motyl T, Moussa CEH, Müller S, Muller S, Münger K, Münz C, Murphy LO, Murphy ME, Musarò A, Mysorekar I, Nagata E, Nagata K, Nahimana A, Nair U, Nakagawa T, Nakahira K, Nakano H, Nakatogawa H, Nanjundan M, Naqvi NI, Narendra DP, Narita M, Navarro M, Nawrocki ST, Nazarko TY, Nemchenko A, Netea MG, Neufeld TP, Ney PA, Nezis IP, Nguyen HP, Nie D, Nishino I, Nislow C, Nixon RA, Noda T, Noegel AA, Nogalska A, Noguchi S, Notterpek L, Novak I, Nozaki T, Nukina N, Nürnberger T, Nyfeler B, Obara K, Oberley TD, Oddo S, Ogawa M, Ohashi T, Okamoto K, Oleinick NL, Oliver FJ, Olsen LJ, Olsson S, Opota O, Osborne TF, Ostrander GK, Otsu K, Ou J-h J, Ouimet M, Overholtzer M, Ozpolat B, Paganetti P, Pagnini U, Pallet N, Palmer GE, Palumbo C, Pan T, Panaretakis T, Pandey UB, Papackova Z, Papassideri I, Paris I, Park J, Park OK, Parys JB, Parzych KR, Patschan S, Patterson C, Pattingre S, Pawelek JM, Peng J, Perlmutter DH, Perrotta I, Perry G, Pervaiz S, Peter M, Peters GJ, Petersen M, Petrovski G, Phang JM, Piacentini M, Pierre P, Pierrefite-Carle V, Pierron G, Pinkas-Kramarski R, Piras A, Piri N, Platanias LC, Pöggeler S, Poirot M, Poletti A, Poüs C, Pozuelo-Rubio M, Prætorius-Ibba M, Prasad A, Prescott M, Priault M, Produit-Zengaffinen N, Progulske-Fox A, Proikas-Cezanne T, Przedborski S, Przyklenk K, Puertollano R, Puyal J, Qian S-B, Qin L, Qin Z-H, Quaggin SE, Raben N, Rabinowich H, Rabkin SW, Rahman I, Rami A, Ramm G, Randall G, Randow F, Rao VA, Rathmell JC, Ravikumar B, Ray SK, Reed BH, Reed JC, Reggiori F, Régnier-Vigouroux A, Reichert AS, Reiners JJ, Reiter RJ, Ren J, Revuelta JL, Rhodes CJ, Ritis K, Rizzo E, Robbins J, Roberge M, Roca H, Roccheri MC, Rocchi S, Rodemann HP, Rodríguez de Córdoba S, Rohrer B, Roninson IB, Rosen K, Rost-Roszkowska MM, Rouis M, Rouschop KMA, Rovetta F, Rubin BP, Rubinsztein DC, Ruckdeschel K, Rucker EB, Rudich A, Rudolf E, Ruiz-Opazo N, Russo R, Rusten TE, Ryan KM, Ryter SW, Sabatini DM, Sadoshima J, Saha T, Saitoh T, Sakagami H, Sakai Y, Salekdeh GH, Salomoni P, Salvaterra PM, Salvesen G, Salvioli R, Sanchez AMJ, Sánchez-Alcázar JA, Sánchez-Prieto R, Sandri M, Sankar U, Sansanwal P, Santambrogio L, Saran S, Sarkar S, Sarwal M, Sasakawa C, Sasnauskiene A, Sass M, Sato K, Sato M, Schapira AHV, Scharl M, Schätzl HM, Scheper W, Schiaffino S, Schneider C, Schneider ME, Schneider-Stock R, Schoenlein PV, Schorderet DF, Schüller C, Schwartz GK, Scorrano L, Sealy L, Seglen PO, Segura-Aguilar J, Seiliez I, Seleverstov O, Sell C, Seo JB, Separovic D, Setaluri V, Setoguchi T, Settembre C, Shacka JJ, Shanmugam M, Shapiro IM, Shaulian E, Shaw RJ, Shelhamer JH, Shen H-M, Shen W-C, Sheng Z-H, Shi Y, Shibuya K, Shidoji Y, Shieh J-J, Shih C-M, Shimada Y, Shimizu S, Shintani T, Shirihai OS, Shore GC, Sibirny AA, Sidhu SB, Sikorska B, Silva-Zacarin ECM, Simmons A, Simon AK, Simon H-U, Simone C, Simonsen A, Sinclair DA, Singh R, Sinha D, Sinicrope FA, Sirko A, Siu PM, Sivridis E, Skop V, Skulachev VP, Slack RS, Smaili SS, Smith DR, Soengas MS, Soldati T, Song X, Sood AK, Soong TW, Sotgia F, Spector SA, Spies CD, Springer W, Srinivasula SM, Stefanis L, Steffan JS, Stendel R, Stenmark H, Stephanou A, Stern ST, Sternberg C, Stork B, Strålfors P, Subauste CS, Sui X, Sulzer D, Sun J, Sun S-Y, Sun Z-J, Sung JJY, Suzuki K, Suzuki T, Swanson MS, Swanton C, Sweeney ST, Sy L-K, Szabadkai G, Tabas I, Taegtmeyer H, Tafani M, Takács-Vellai K, Takano Y, Takegawa K, Takemura G, Takeshita F, Talbot NJ, Tan KSW, Tanaka K, Tanaka K, Tang D, Tang D, Tanida I, Tannous BA, Tavernarakis N, Taylor GS, Taylor GA, Taylor JP, Terada LS, Terman A, Tettamanti G, Thevissen K, Thompson CB, Thorburn A, Thumm M, Tian F, Tian Y, Tocchini-Valentini G, Tolkovsky AM, Tomino Y, Tönges L, Tooze SA, Tournier C, Tower J, Towns R, Trajkovic V, Travassos LH, Tsai T-F, Tschan MP, Tsubata T, Tsung A, Turk B, Turner LS, Tyagi SC, Uchiyama Y, Ueno T, Umekawa M, Umemiya-Shirafuji R, Unni VK, Vaccaro MI, Valente EM, Van den Berghe G, van der Klei IJ, van Doorn W, van Dyk LF, van Egmond M, van Grunsven LA, Vandenabeele P, Vandenberghe WP, Vanhorebeek I, Vaquero EC, Velasco G, Vellai T, Vicencio JM, Vierstra RD, Vila M, Vindis C, Viola G, Viscomi MT, Voitsekhovskaja OV, von Haefen C, Votruba M, Wada K, Wade-Martins R, Walker CL, Walsh CM, Walter J, Wan X-B, Wang A, Wang C, Wang D, Wang F, Wang F, Wang G, Wang H, Wang H-G, Wang H-D, Wang J, Wang K, Wang M, Wang RC, Wang X, Wang X, Wang Y-J, Wang Y, Wang Z, Wang ZC, Wang Z, Wansink DG, Ward DM, Watada H, Waters SL, Webster P, Wei L, Weihl CC, Weiss WA, Welford SM, Wen L-P, Whitehouse CA, Whitton JL, Whitworth AJ, Wileman T, Wiley JW, Wilkinson S, Willbold D, Williams RL, Williamson PR, Wouters BG, Wu C, Wu D-C, Wu WKK, Wyttenbach A, Xavier RJ, Xi Z, Xia P, Xiao G, Xie Z, Xie Z, Xu D-z, Xu J, Xu L, Xu X, Yamamoto A, Yamamoto A, Yamashina S, Yamashita M, Yan X, Yanagida M, Yang D-S, Yang E, Yang J-M, Yang SY, Yang W, Yang WY, Yang Z, Yao M-C, Yao T-P, Yeganeh B, Yen W-L, Yin J-j, Yin X-M, Yoo O-J, Yoon G, Yoon S-Y, Yorimitsu T, Yoshikawa Y, Yoshimori T, Yoshimoto K, You HJ, Youle RJ, Younes A, Yu L, Yu L, Yu S-W, Yu WH, Yuan Z-M, Yue Z, Yun C-H, Yuzaki M, Zabirnyk O, Silva-Zacarin E, Zacks D, Zacksenhaus E, Zaffaroni N, Zakeri Z, Zeh HJ, Zeitlin SO, Zhang H, Zhang H-L, Zhang J, Zhang J-P, Zhang L, Zhang L, Zhang M-Y, Zhang XD, Zhao M, Zhao Y-F, Zhao Y, Zhao ZJ, Zheng X, Zhivotovsky B, Zhong Q, Zhou C-Z, Zhu C, Zhu W-G, Zhu X-F, Zhu X, Zhu Y, Zoladek T, Zong W-X, Zorzano A, Zschocke J, Zuckerbraun B (2012). Guidelines for the use and interpretation of assays for monitoring autophagy. Autophagy.

[21] Chen SH, Zhang BY, Zhou B, Zhu CZ, Sun LQ, Feng YJ (2019). Perineural invasion of cancer: a complex crosstalk between cells and molecules in the perineural niche. Am J Cancer Res.

[22] Schorn S, Demir IE, Haller B, Scheufele F, Reyes CM, Tieftrunk E, Sargut M, Goess R, Friess H, Ceyhan GO (2017). The influence of neural invasion on survival and tumor recurrence in pancreatic ductal adenocarcinoma - A systematic review and meta-analysis. Surg Oncol.

[23] Crippa S, Pergolini I, Javed AA, Honselmann KC, Weiss MJ, Di Salvo F, et al. Implications of Perineural Invasion on Disease Recurrence and Survival After Pancreatectomy for Pancreatic Head Ductal Adenocarcinoma. Ann Surg. 2020.10.1097/SLA.0000000000004464. Epub ahead of print. 10.1097/SLA.0000000000004464 33086324

[24] Gasparini G, Pellegatta M, Crippa S, Lena MS, Belfiori G, Doglioni C, et al. Nerves and Pancreatic Cancer: New Insights into a Dangerous Relationship. Cancers (Basel). 2019;11(7):893. 10.3390/cancers11070893 PMC6678884 31248001

[25] Kuol N, Stojanovska L, Apostolopoulos V, Nurgali K (2018). Role of the nervous system in cancer metastasis. J Exp Clin Cancer Res.

[26] Faulkner S, Jobling P, March B, Jiang CC, Hondermarck H (2019). Tumor Neurobiology and the War of Nerves in Cancer. Cancer Discov.

[27] Saloman JL, Albers KM, Li D, Hartman DJ, Crawford HC, Muha EA, Rhim AD, Davis BM (2016). Ablation of sensory neurons in a genetic model of pancreatic ductal adenocarcinoma slows initiation and progression of cancer. Proc Natl Acad Sci U S A.

[28] Hutchings C, Phillips JA, Djamgoz MBA (2020). Nerve input to tumours: Pathophysiological consequences of a dynamic relationship. Biochim Biophys Acta Rev Cancer.

[29] Chavez-Dominguez R, Perez-Medina M, Lopez-Gonzalez JS, Galicia-Velasco M, Aguilar-Cazares D (2020). The Double-Edge Sword of Autophagy in Cancer: From Tumor Suppression to Pro-tumor Activity. Front Oncol.

[30] Nawrocki ST, Wang W, Carew JS. Autophagy: New Insights into Its Roles in Cancer Progression and Drug Resistance. Cancers (Basel). 2020;12(10):3005. 10.3390/cancers12103005 PMC7602821 33081217

[31] Ho CJ, Gorski SM. Molecular Mechanisms Underlying Autophagy-Mediated Treatment Resistance in Cancer. Cancers (Basel). 2019;11(11):1775. 10.3390/cancers11111775 PMC6896088 31717997

[32] Yang S, Wang X, Contino G, Liesa M, Sahin E, Ying H, Bause A, Li Y, Stommel JM, Dell'antonio G, Mautner J, Tonon G, Haigis M, Shirihai OS, Doglioni C, Bardeesy N, Kimmelman AC (2011). Pancreatic cancers require autophagy for tumor growth. Genes Dev.

[33] Piffoux M, Eriau E, Cassier PA (2021). Autophagy as a therapeutic target in pancreatic cancer. Br J Cancer.

[34] Endo S, Nakata K, Ohuchida K, Takesue S, Nakayama H, Abe T, Koikawa K, Okumura T, Sada M, Horioka K, Zheng B, Mizuuchi Y, Iwamoto C, Murata M, Moriyama T, Miyasaka Y, Ohtsuka T, Mizumoto K, Oda Y, Hashizume M, Nakamura M (2017). Autophagy Is Required for Activation of Pancreatic Stellate Cells, Associated With Pancreatic Cancer Progression and Promotes Growth of Pancreatic Tumors in Mice. Gastroenterology.

[35] Yang YH, Liu JB, Gui Y, Lei LL, Zhang SJ (2017). Relationship between autophagy and perineural invasion, clinicopathological features, and prognosis in pancreatic cancer. World J Gastroenterol.

[36] Demir IE, Boldis A, Pfitzinger PL, Teller S, Brunner E, Klose N, et al. Investigation of Schwann cells at neoplastic cell sites before the onset of cancer invasion. J Natl Cancer Inst. 2014;106(8):dju184. 10.1093/jnci/dju184 25106646

[37] Su D, Guo X, Huang L, Ye H, Li Z, Lin L, Chen R, Zhou Q (2020). Tumor-neuroglia interaction promotes pancreatic cancer metastasis. Theranostics.

[38] Endo T, Kadoya K, Kawamura D, Iwasaki N (2019). Evidence for cell-contact factor involvement in neurite outgrowth of dorsal root ganglion neurons stimulated by Schwann cells. Exp Physiol.

[39] Gao D, Tang T, Zhu J, Tang Y, Sun H, Li S (2019). CXCL12 has therapeutic value in facial nerve injury and promotes Schwann cells autophagy and migration via PI3K-AKT-mTOR signal pathway. Int J Biol Macromol.

[40] Zhu Z, Friess H, di Mola FF, Zimmermann A, Graber HU, Korc M, Buchler MW (1999). Nerve growth factor expression correlates with perineural invasion and pain in human pancreatic cancer. J Clin Oncol.

[41] Ceyhan GO, Schafer KH, Kerscher AG, Rauch U, Demir IE, Kadihasanoglu M, Bohm C, Muller MW, Buchler MW, Giese NA, Erkan M, Friess H (2010). Nerve growth factor and artemin are paracrine mediators of pancreatic neuropathy in pancreatic adenocarcinoma. Ann Surg.

[42] Ma J, Jiang Y, Jiang Y, Sun Y, Zhao X (2008). Expression of nerve growth factor and tyrosine kinase receptor A and correlation with perineural invasion in pancreatic cancer. J Gastroenterol Hepatol.

[43] Jiang J, Bai J, Qin T, Wang Z, Han L (2020). NGF from pancreatic stellate cells induces pancreatic cancer proliferation and invasion by PI3K/AKT/GSK signal pathway. J Cell Mol Med.

[44] Hu J, Zhou J, Li X, Wang F, Lu H (2011). Schwann cells promote neurite outgrowth of dorsal root ganglion neurons through secretion of nerve growth factor. Indian J Exp Biol.

[45] Wolpin BM, Rubinson DA, Wang X, Chan JA, Cleary JM, Enzinger PC, Fuchs CS, McCleary NJ, Meyerhardt JA, Ng K, Schrag D, Sikora AL, Spicer BA, Killion L, Mamon H, Kimmelman AC (2014). Phase II and pharmacodynamic study of autophagy inhibition using hydroxychloroquine in patients with metastatic pancreatic adenocarcinoma. Oncologist.

[46] Lei Y, Tang L, Xie Y, Xianyu Y, Zhang L, Wang P, Hamada Y, Jiang K, Zheng W, Jiang X (2017). Gold nanoclusters-assisted delivery of NGF siRNA for effective treatment of pancreatic cancer. Nat Commun.

[47] Reavis HD, Chen HI, Drapkin R (2020). Tumor Innervation: Cancer Has Some Nerve. Trends Cancer.

[48] Saloman JL, Singhi AD, Hartman DJ, Normolle DP, Albers KM, Davis BM (2018). Systemic Depletion of Nerve Growth Factor Inhibits Disease Progression in a Genetically Engineered Model of Pancreatic Ductal Adenocarcinoma. Pancreas.

[49] Bapat AA, Munoz RM, Von Hoff DD, Han H (2016). Blocking Nerve Growth Factor Signaling Reduces the Neural Invasion Potential of Pancreatic Cancer Cells. PLoS One.

