# Supplementary figures and images for: Autophagic Schwann cells promote perineural invasion mediated by the NGF/ATG7 paracrine pathway in pancreatic cancer
Source: J Exp Clin Cancer Res. 2022 Feb 2;41:48. doi: 10.1186/s13046-021-02198-w (PMC8809009; doi:10.1186/s13046-021-02198-w)

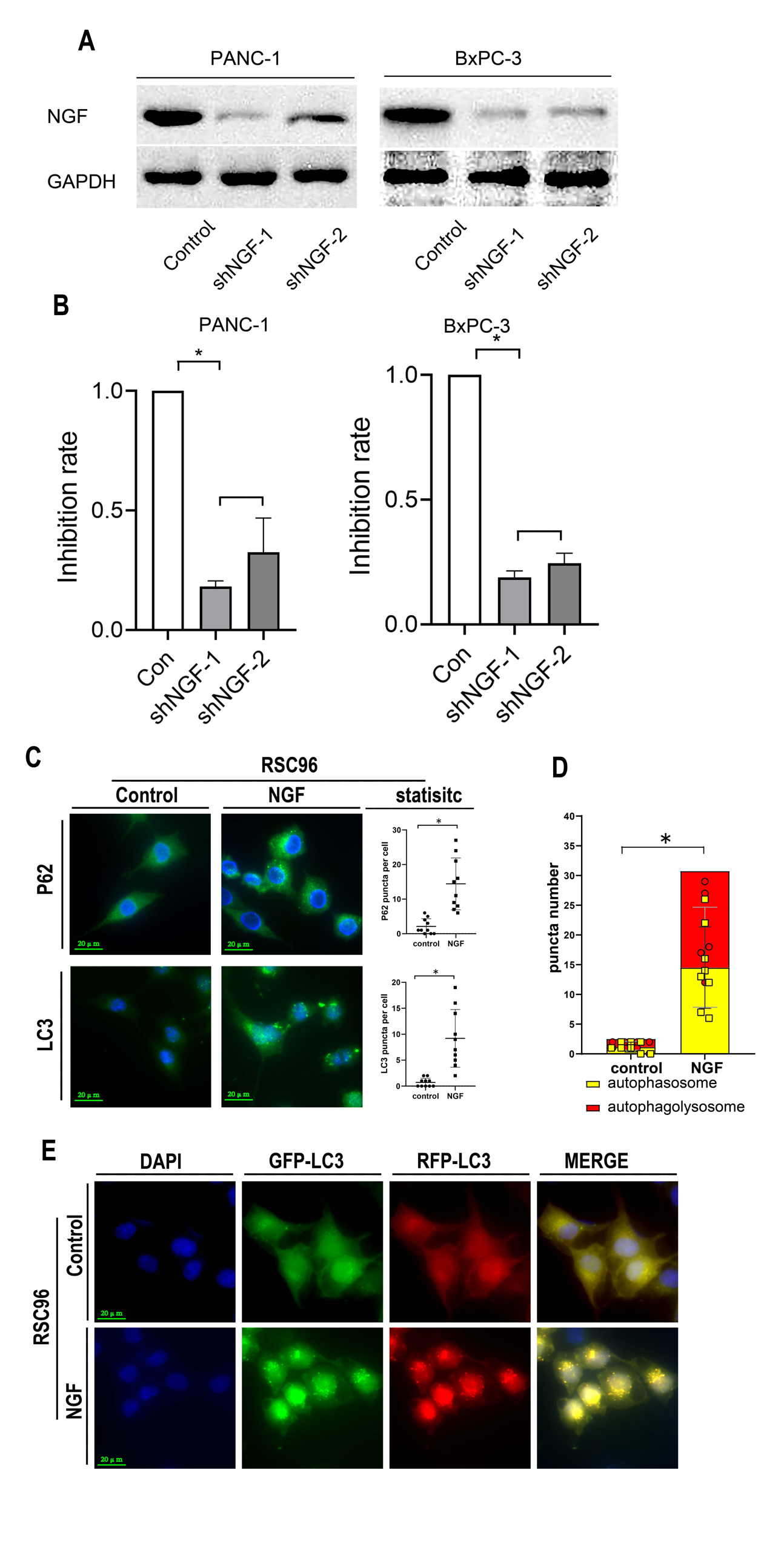

Supplement: Supplementary file 2 — Additional file 2: Fig. S2. A. Western blotting of NGF in the control or NGF knockdown PDAC cell lines. GAPDH was used as a loading control. B. NGF protein expression in PanCa and shNGF-PanCa cell lines in PANC-1 and BxPC-3 cell lines. C. NGF (50 ng/ml) induces the P62 and LC3 puncta accumulation in RSC96 cell line. D. Statistic of autophasosomes and autophagolysosomes in RSC96 treated with NGF (50 ng/ml) or control. (cell image: Fig. S2E). E. Autophagic flux detection of RSC96 cells treated with the control or NGF (50 ng/ml). NGF promotes autophagy flux in RSC96 cells. [file 13046_2021_2198_MOESM2_ESM.jpg]

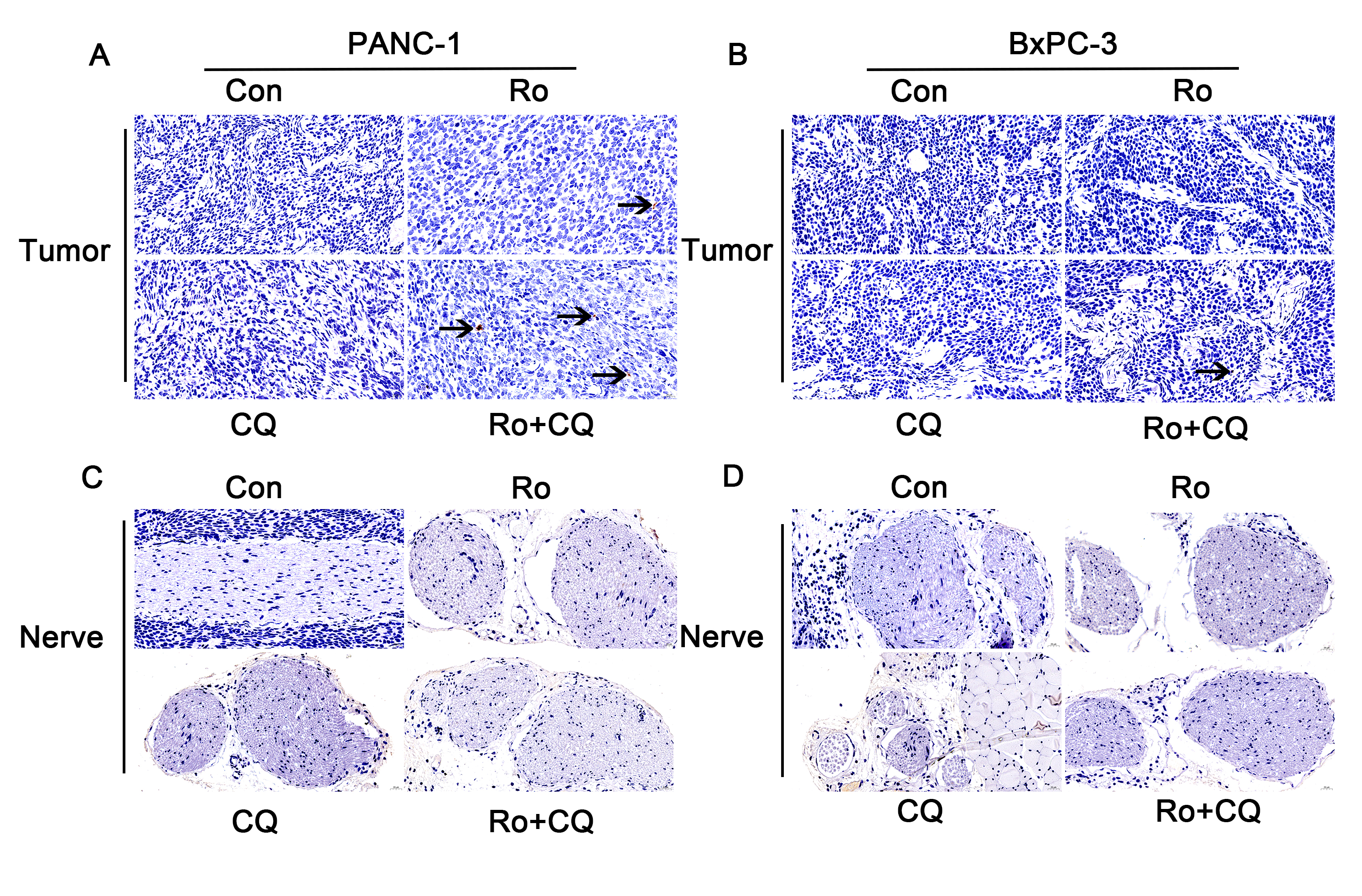

Supplement: Supplementary file 7 — Additional file 7: Fig. S7. A. Cleaved caspase-3 IHC staining of tumors in the PANC-1 nerve invasion model treated with the control, RO, CQ or RO + CQ. Black arrow indicates cleaved caspase-3-positive cancer cells. B. Cleaved caspase-3 IHC staining of tumors in the BxPC-3 nerve invasion model treated with the control, RO, CQ or RO + CQ. Black arrow indicates cleaved caspase-3-positive cancer cells. C. Cleaved caspase-3 IHC staining of nerves in the PANC-1 nerve invasion model treated with the control, RO, CQ or RO + CQ. D. Cleaved caspase-3 IHC staining of nerves in the BxPC-3 nerve invasion model treated with the control, RO, CQ or RO + CQ. [file 13046_2021_2198_MOESM7_ESM.jpg]
